# Nuclear Factor I Family Members are Key Transcription Factors Regulating Gene Expression

**DOI:** 10.1016/j.mcpro.2024.100890

**Published:** 2024-11-29

**Authors:** Dicle Malaymar Pinar, Helka Göös, Zenglai Tan, Esa-Pekka Kumpula, Iftekhar Chowdhury, Zixian Wang, Qin Zhang, Kari Salokas, Salla Keskitalo, Gong-Hong Wei, Asli Kumbasar, Markku Varjosalo

**Affiliations:** 1 Institute of Biotechnology, HiLIFE, University of Helsinki, Helsinki, Finland; 2 Department of Molecular Biology and Genetics, Istanbul Technical University, Istanbul, Turkey; 3 iCell, Research and Development, Finnish Red Cross Blood Service, Helsinki, Finland; 4 Biocenter Oulu and Faculty of Biochemistry and Molecular Medicine, University of Oulu, Oulu, Finland; 5 MOE Key Laboratory of Metabolism and Molecular Medicine & Department of Biochemistry and Molecular Biology of School Basic Medical Sciences, Fudan University Shanghai Cancer Center, Shanghai Medical College of Fudan University, Shanghai, China; 6 iCAN Digital Precision Cancer Medicine Flagship, Faculty of Medicine, University of Helsinki, Helsinki, Finland

**Keywords:** nuclear factor I, transcription factors, affinity purification, proximity-dependent biotinylation, BioID, protein-protein interactions, transcriptional regulation, interaction proteomics, mass spectrometry, ChIP-seq

## Abstract

The Nuclear Factor I (NFI) family of transcription factors (TFs) plays key roles in cellular differentiation, proliferation, and homeostasis. As such, NFI family members engage in a large number of interactions with other proteins and chromatin. However, despite their well-established significance, the NFIs’ interactomes, their dynamics, and their functions have not been comprehensively examined. Here, we employed complementary omics-level techniques, *i.e.* interactomics (affinity purification mass spectrometry (AP-MS) and proximity-dependent biotinylation (BioID)), and chromatin immunoprecipitation sequencing (ChIP-Seq), to obtain a comprehensive view of the NFI proteins and their interactions in different cell lines. Our analyses included all four NFI family members, and a less-studied short isoform of NFIB (NFIB4), which lacks the DNA binding domain. We observed that, despite exhibiting redundancy, each family member had unique high-confidence interactors and target genes, suggesting distinct roles within the transcriptional regulatory networks. The study revealed that NFIs interact with other TFs to co-regulate a broad range of regulatory networks and cellular processes. Notably, time-dependent proximity-labeling unveiled a highly dynamic nature of NFI protein-protein interaction networks and hinted at the temporal modulation of NFI interactions. Furthermore, gene ontology (GO) enrichment analysis of NFI interactome and targetome revealed the involvement of NFIs in transcriptional regulation, chromatin organization, cellular signaling pathways, and pathways related to cancer. Additionally, we observed that NFIB4 engages with proteins associated with mRNA regulation, which suggests that NFIs have roles beyond traditional DNA binding and transcriptional modulation. We propose that NFIs may function as potential pioneering TFs, given their role in regulating the DNA binding ability of other TFs and their interactions with key chromatin remodeling complexes, thereby influencing a wide range of cellular processes. These insights into NFI protein-protein interactions and their dynamic, context-dependent nature provide a deeper understanding of gene regulation mechanisms and hint at the role of NFIs as master regulators.

## Highlights

• AP-MS, BioID, and ChIP-Seq techniques used for identifying NFI regulatory networks.
• NFIs have both overlapping and distinct functions in the transcriptional regulation.
• NFIA co-regulates gene expression with SOX2.
• NFIA can affect the DNA binding of SOX2.
• NFIs interactome and targetome share transcription regulation and cancer-related terms.
• NFIB4, a NFI isoforms lacking DBD, may have roles in post-transcriptional processes.

## Graphical Abstract

**Figure:**
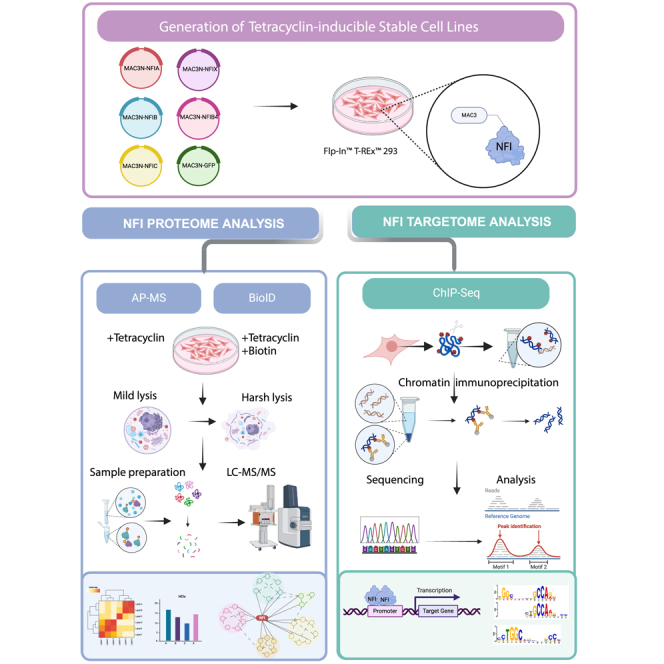

## In Brief

This study reveals the Nuclear Factor I (NFI) family as key transcription factors that regulate transcription, chromatin organization, and cellular signaling. Using comprehensive omics approaches, we identified unique and dynamic interactions among NFI proteins, including an alternative splicing isoform lacking DNA binding ability, NFIB4. Our findings highlight NFIs as master regulators, orchestrating gene expression and influencing key cellular processes, with significant implications for understanding transcriptional regulation and potential roles in cancer.

Although originally identified more than 40 years ago as nuclear proteins involved in adenovirus DNA replication (1), members of the nuclear factor I (NFI) family of transcription factors (TFs) are now recognized as key regulators of cell proliferation, stem cell fate specification and differentiation, and tumorigenesis (2, 3, 4, 5). In vertebrates, the NFI family consists of four members: NFIA, NFIB, NFIC, and NFIX. While each NFI gene encodes various alternative splicing isoforms, the canonical NFI isoforms share a highly conserved N-terminal DNA binding and dimerization domain (DBD), and a more variable C-terminal region, which is responsible for activating or repressing gene expression (6). All members of the NFI family bind to the “TTGGC(N5)GCCAA” DNA motif with similar affinity, as homo- or heterodimers, potentially regulating the same set of target genes and implying functional redundancy (7, 8). The functional redundancy is further supported by the fact that NFIs can substitute for each other as shown by the similarity of double and heterozygous knockout phenotypes (6, 9). However, distinct phenotypes in specific tissues and developmental stages have also been observed and support specific and unique roles for NFIA, NFIB, NFIX in the central nervous system (8, 10, 11, 12), NFIB in lung development (13, 14) and NFIC in tooth and bone formation (15, 16, 17). Moreover, different family members can play divergent roles in disease even within the same cancer type. For example, while both NFIA and NFIB are required for *in vitro* and *in vivo* glioblastoma growth (18), NFIA overexpression leads to cell proliferation and migration, whereas NFIB suppresses tumor growth and promotes glial differentiation (19, 20, 21). Therefore, despite binding to the same DNA motif with similar affinity, each family member executes specific functions, suggesting that this specificity is likely determined by factors other than DNA sequence specificity.

Previous studies have indicated that NFI gene regulation specificity could be achieved by distinct expression patterns, alternative splicing, and/or by engaging with different subsets of other transcriptional regulators and co-factors via isoform-specific C-terminal regions (22, 23, 24). For example, our previous work on human TF interaction networks revealed that NFI members have a surprising ability to engage with other TFs, as they are involved in more than half (118 out of 202) of all TF-TF interactions we detected. Importantly, NFIs bind and functionally regulate major transcription factor families such as SOXs, KLFs, and PAXs (25). In the case of NFIA, this was in agreement with previously reported NFIA/SOX9 and NFIA/SOX10 interactions, which have opposing effects in the initiation of gliogenesis and astrocyte fate decision (26, 27, 28). However, although some interaction partners and functional consequences of TF-TF interactions have been previously studied, many details remain unresolved, especially in the context of understanding how NFIs employ protein-protein interactions to govern their chromatin binding specificity and gene regulation.

To address this, here we conducted a comprehensive investigation of the interactors and targets of the NFI family of TFs using advanced multiomics techniques such as affinity purification mass spectrometry (AP-MS), proximity labeling, and chromatin immunoprecipitation sequencing (ChIP-Seq). Our analyses identified common interactors and targets that underline the basis for functional redundancy of NFI members. Importantly, we also detected many specific high-confidence interactions (HCIs) and downstream targets connected with distinct roles of individual NFIs. Furthermore, our research across various cell lines revealed that NFIs interact with both shared and cell type-specific proteins, indicating their cellular context-dependent roles. Moreover, we used time-dependent interactome analysis that allowed us to examine the dynamics of NFI interactome, revealing how interactome changes over time and how NFI-mediated regulation propagates through gene regulatory networks. Of note, this study represents the first in-depth analysis of the NFIB4 isoform, and we showed that it engages with proteins associated with mRNA regulation suggesting a role in post-transcriptional processes. Taken together, our findings place NFI members as key regulators within many gene regulatory networks where they exert dynamic and context-dependent functions.

## Experimental Procedures

### Experimental Design and Statistical Rationale

All experiments performed in this manuscript were conducted as replicates. For AP-MS and BioID experiments, NFI fusion protein-expressing cell lines were generated as biological triplicates for each bait. We included MAC3-N-tagged GFP expressing cell line as six replicates for BioID samples, and three replicates for AP-MS experiments to filter out nonspecific interactors. The same stable cell lines were used for BioID experiments in two different biotinylation times. Western blotting and localization images are representative of results from a minimum of three independent experiments. The total protein concentration of lysates was measured and equalized for blotting. Chromatin immunoprecipitation experiments were performed with the same stable NFI fusion cell lines generated for AP and BioID experiments. Reporter assay experiments were performed as triplicates.

For gene ontology (GO) term enrichment analysis, terms with Benjamini (adjusted *p*-value) values less than 0.001 are considered as enriched. KEGG pathways with FDR <0.05 is used for generating dot plot figures of ChIP-Seq results.

### Cell Culture

In this study, the Flp-In transduction system using Flp-In T-REx 293 cell line (Invitrogen, Cat# R78007), allowing to generate isogenic and inducible stable cell clones with only a single copy of a transgene in their genome, provides a robust method to study PPIs (29). U2OS (ATCC, HTB-96) cells were used to visualize protein localization in the cell via fluorescence microscopy. Cells were cultured using low glucose DMEM (Sigma Aldrich) supplemented with 10% FBS and 100 μg/ml penicillin/streptomycin (Life Technologies) at 37 °C with 5% CO_2_.

SH-SY5Y neuroblastoma cell line (ATCC, CRL-2266) was used for lentiviral transduction of full-length NFIB and short isoform NFIB4 encoding constructs. SH-SY5Y cells cultured in Dulbecco's modified Eagle's medium: Nutrient mixture F12 (DMEM/F12) (Gibco) supplemented with 10% FBS, 1% 100 μg/ml penicillin/streptomycin (Life Technologies) at 37 °C with 5% CO_2_. U251-MG glioblastoma cell line was used for transient transfection of MAC3-N-tagged NFIA, NFIC, NFIB4, and GFP constructs. Cells were cultured in DMEM (Sigma Aldrich) supplemented with 10% FBS and 100 μg/ml penicillin/streptomycin (Life Technologies) at 37 °C with 5% CO_2_.

### Generation of Stable, Inducible MAC3-tagged NFI Expressing Cell Lines

Full-length NFIA, NFIB, NFIC, and NFIX constructs from the human ORFeome collections and short NFIB isoform, NFIB4, were used for this study. Using Gateway cloning, NFI coding sequences without stop codons were cloned into the pDONR221 entry vector. To generate tetracycline-inducible stable cell lines, constructs were cloned into N-terminal (MAC3-tag-N) destination vector and introduced into Flp-In T-REx 293 cells (Life Technologies, Carlsbad, CA) to generate stable, isogenic, and inducible cell lines as described by Liu *et al.* (29).

### Generation of Stable SH-SY5Y Cell Lines by Lentiviral Transduction

To generate lentiviral constructs, N-terminally myc-BioID2 tagged NFIB and NFIB4 sequences were cloned into pENTR1A entry vector by In-Fusion HD Cloning Kit according to the manufacturer’s instructions. pENTR1A linearized with BamHI/XbaI double enzyme digestion and fusion protein coding sequences amplified by PCR with 15-bp homologous extension in primers. PCR products and linearized entry vector purified and ligated by In-fusion reaction. Verified constructs were transferred into pLenti_CMVNeo/DEST destination vector by Gateway cloning.

### Lentivirus Packaging and Production

According to the protocol of Campeau *et al.* (30), HEK293T cells were transfected with third-generation packaging vectors (pMD2.G, pMDLg/pRRE, pRSV-Rev) and pLenti_CMVNeo/Dest vectors carrying each constructs (myc-BioID2-NFIB3, myc-BioID2-NFIB4, myc-BioID2). Media containing lentiviral particles were collected at 48 and 72 h after transfection. The media collected for the precipitation of viruses were kept in 10% PEG overnight, and the next day, the mixture was centrifuged at +4 °C, 3600g for 1 h. The supernatant was discarded and the resulting virus pellet was suspended in the remaining 1 ml medium.

On the day before transduction, 1.5 × 10^5^ SH-SY5Y cells were seeded in 35 mm petri dishes. The following day, virus samples were added along with 1 ml of media containing 8 μg/ml of Protamine Sulfate. For selection, 600 μg/ml G418 was added 24 h after transduction. Media containing G418 was changed every 2 to 3 days, and the cells were expanded to obtain required amount (1.5 × 10^7^) of cells.

### Transient Transfection of U251-MG Glioblastoma Cell Line

To generate cell lines expressing MAC3-tagged NFI proteins, constructs were transfected into U251-MG cells using the Invitrogen Neon Transfection System.

For each construct, 3 × 10^7^ glioblastoma cells were harvested and resuspended in a Neon Transfection Buffer. For each replicate, 1 × 10^7^ cells and 50 μg of NFI-MAC3N constructs were aliquoted into a sterile tube (500 μl total volume). Cells were pulsed 5 times (5 × 100 μl) and collected in 10 ml antibiotic-free complete media. Electroporation parameters were applied as 1150 V, 30 ms width, and one pulse for U251-MG cells.

The cell suspension was added to prewarm 20 ml antibiotic-free complete media containing 15 cm plates. The next day, transfection efficiency and viability were checked with GFP-transfected cells, and 50 μM biotin was added to plates. Cells were harvested 16 h after biotin addition.

### Western Blotting

Following harsh lysis and sonication of BioID samples, a small amount of cell lysate was kept for the Western blotting experiment. Total protein concentration determined by BCA assay according to manufacturer instructions. For the Western blot experiment, ∼15 μg (5 μl) lysate was mixed with 5× SDS Sample Buffer and incubated at 95 °C for 5 min. Denatured protein samples were loaded onto 12% SDS PAGE gels. Proteins were transferred onto nitrocellulose membrane using Semidry System (BIORAD) following PAGE. After transfer, the membrane was blocked with 5% non-fat milk powder in TBS-T for 1 h at room temperature with gentle shaking. Then, anti-HA (1:2000) primary antibody solution was applied and the membrane was incubated overnight at +4 °C on a rotating shaker. The next day, after washing with TBS-T, and the membrane was incubated in a secondary antibody solution for 1 h at room temperature with gentle shaking. Proteins were visualized with Amersham ECL Prime Western Blotting Detection Reagent using iBright Imaging Systems (Thermo Fisher). After the detection of the proteins, the membrane was stripped by incubating with Restore PLUS Western Blot Stripping Buffer for 15 min. Membrane wash with TBS-T and blocked with 5% non-fat milk powder in TBS-T for 1 h at room temperature. Then alpha-tubulin antibody (1:1000) solution was applied and the same protocol for incubation and visualization was repeated as described above.

### Affinity Pull-Down and Dot Blot Analysis

To validate SWI/SNF proteins and NFI interactions, HEK293 cells (5 × 10⁵ per well) were seeded in a 6-well plate and co-transfected with 500 ng of HA-tagged bait construct and 500 ng of V5-tagged prey construct using Fugene six transfection reagent (Promega). After 48 h, cells were washed with ice-cold PBS and lysed using 1 ml of HENN buffer (50 mM HEPES pH 8.0, 5 mM EDTA, 150 mM NaCl, 50 mM NaF, 0.5% IGEPAL, 1 mM DTT, 1 mM PMSF, 1.5 mM Na₃VO₄, and a protease inhibitor cocktail). Lysates were collected and cleared by centrifugation at 16,000*g* for 20 min at 4 °C. Next, 30 μl of Strep-Tactin Sepharose resin (IBA Lifesciences GmbH) was prepared by washing it twice with 200 μl of HENN buffer (4000*g*, 1 min, 4 °C). The cleared lysate was then incubated with the pre-washed Strep-Tactin beads for 1 h at 4 °C on a rotation wheel. The beads were collected by centrifugation (4000*g*, 30 s, 4 °C) and washed 3 times with 1 ml of HENN buffer. After the final wash, the beads were resuspended in 60 μl of 2 × Laemmli sample buffer (Bio-Rad) and boiled for 5 min at 95 °C.

For the dot blot, 10 μl of each sample was spotted onto a pre-washed nitrocellulose membrane using the Bio-Dot Microfiltration System (Bio-Rad). The membrane was blocked with 5% milk in TBS-T (0.05% Tween-20 in TBS) for 1 h at room temperature, then incubated with a primary antibody targeting prey proteins (mouse anti-V5, 1:5000 dilution) overnight at 4 °C. After three washes with TBS-T, the membrane was incubated with an HRP-conjugated secondary antibody (goat anti-mouse IgG, 1:2000 dilution) for 1 h at room temperature. Following additional washes, the membrane was treated with ECL Prime detection reagent (Cytiva) and visualized using iBright Imaging Systems (Thermo Fisher). To detect the bait protein, the membrane was stripped with Restore Plus Stripping Buffer (Thermo Fisher), reblocked, and incubated overnight at 4 °C with a second primary antibody targeting NFIs (rabbit anti-HA, 1:2000 dilution). After repeating the washing and secondary antibody incubation steps, the membrane was visualized as described earlier.

### Immunocytochemistry

The localization of the MAC3-NFI proteins was examined in U2OS cells. The day before transfection, 1.5 × 10^5^ U2OS cells were plated in a 6-well plate. The next day, approximately 3 × 10^5^ cells with 2 μg plasmid and 6 μl Fugene 6 Transfection Reagent. After 30 min incubation of transfection reaction, the solution was added dropwise to U2OS cells and mixed gently by rocking the plate back and forth. Plates were incubated at 37 °C, 5% CO_2_ incubator for 24 h. After 24 h, the culture medium was removed, and cells were washed with PBS. Cells were trypsinized, neutralized with DMEM Full, and pelleted by centrifugation at 300*g* for 5 min. The pellet was resuspended 1.2 ml DMEM to have 50.000 cells/100 μl.

5 × 10^4^ cells (100 μl) were seeded on sterilized coverslips. Coverslips are incubated for 3 h in a Petri dish and then they are transferred to the 24-well plates. Biotin-added DMEM was added to 24 well plates containing coverslips. After 16h incubation with biotin-containing media, cells were fixed in 4% paraformaldehyde (PFA) for 15 min and followed by washing with 1× PBS twice. Cells were permeabilized with 0.1% Triton X-100 in PBS for 4 min and blocking 0.2% BSA in DPBS for 20 min at room temperature. After 30 min incubation with primary antibody (anti-HA, 1:500), cells were incubated with Alexa fluor 488 (1:500) (for MAC3-tagged proteins), Alexa fluor 594 (1:1000) (to detect biotinylated proteins) and DAPI (1:1000). Images were obtained with Leica SP8 STED Confocal microscopy.

### NFIA Silencing and Chromatin Immunoprecipitation Sequencing (ChIP-Seq)

NFIA expressing Flp-In T-REx 293 cells were seeded in 10 cm dishes; 24 h later, tetracycline with a final concentration of 2 μg/ml was added to induce the expression of bait protein. siRNAs against NFIA or control were transfected with a final concentration of 100 nM by the lipofectamine RNAiMAX reagent. The ChIP-Seq assays were performed as previously described (31). In brief, HEK293 cells were passaged to 15 cm dishes and treated with tetracycline at a final concentration of 2 μg/ml to induce the expression of TF protein 24 h before harvest. Then cross-linking the cells with 1% formaldehyde for 10 min at room temperature, with the reaction quenched using 125 mM glycine. Following two washes with pre-cooled PBS, cells were lysed in hypotonic lysis buffer (20 mM Tris-Cl, pH 8.0, with 10% glycerol, 10 mM KCl, 2 mM DTT, and complete protease inhibitor cocktail (Roche)) to isolate nuclei. The nuclear pellets were subsequently washed in pre-cooled PBS and lysed in an equal mixture of SDS lysis buffer (50 mM Tris-HCl, pH 8.1, with 1% SDS, 10 mM EDTA, and complete protease inhibitor) and ChIP dilution buffer (16.7 mM Tris-HCl, pH 8.1, with 0.01% SDS, 1.1% Triton X-100, 1.2 mM EDTA, 167 mM NaCl and complete protease inhibitor). Chromatin was sheared to an average size of 300 bp using a Q800R sonicator (QSonica) at 4 °C. Dynabead protein G (Invitrogen), pre-washed with blocking buffer (0.5% BSA in IP buffer (20 mM Tris-HCl, pH 8.0, with 2 mM EDTA, 150 mM NaCl, 1% Triton X-100, and protease inhibitor cocktail)), was incubated with an anti-HA antibody (ab18181, Abcam). The chromatin lysate was incubated with the antibody-coated beads for 12 h and washed 4 times with washing buffer (50 mM HEPES, pH 7.6, 1 mM EDTA, 0.7% sodium deoxycholate, 1% NP-40, 0.5 M LiCl) and twice with 100 mM ammonium hydrogen carbonate (AMBIC) solution. DNA-protein complexes were eluted using extraction buffer (10 mM Tris-HCl, pH 8.0, with 1 mM EDTA and 1% SDS), and proteinase K and NaCl were added to reverse the cross-links. The DNA was purified using a Mini-Elute PCR Purification Kit (Qiagen). The purified DNA was subjected to ChIP-Seq library preparation and sequencing.

### ChIP-Seq Library Processing

Quality control assessments of the ChIP libraries' sequencing results were conducted via FastQC (v0.11.9), scrutinizing the integrity of sequencing data. Adaptors and undersized reads were excised utilizing TrimGalore (v0.6.7, RRID: SCR_011847). Following this, the refined data were aligned to the human reference genome (hg38) using Bowtie2 (v2.2.5) (32) under standard settings. SAMtools (v1.9) (33) was employed to filter out low-quality alignments, applying criteria “-q 30 -F 3844.” Duplicate reads were identified and removed with the Picard toolkit (v2.25.1, RRID: SCR_006525). MACS2 (v2.1.4) (34) facilitated peak calling, adopting a significance threshold of *P* < 1e-3. Peak annotations were executed through the HOMER perl script “annotatePeaks.pl” (v4.11.1) (35).

### Characterization of ChIP-Seq Signal Features

Peak intersections were delineated using the ‘findOverlapsOfPeaks’ function from the ChIPpeakAnno package (v3.24.2), recognizing an overlap when at least one base is shared. The aggregate of peak intersections reflects the combined peak counts across groups, with individual group counts sequentially listed in parentheses. The deepTools suite's “computeMatrix” (v3.5.2) (36) facilitated visualization of ChIP-Seq signal intensities across targeted genomic regions, while “plotHeatmap” was used to generate heatmaps illustrating signal dispersion.

### Pathway and Enrichment Analyses

Utilization of the “clusterProfiler” package (v4.3.0.991) (37) enabled Gene Ontology (GO) and Kyoto Encyclopedia of Genes and Genomes (KEGG) pathway enrichment analyses. The enrichGO and enrichKEGG functions were pivotal in determining significant GO terms and KEGG pathways, adhering to an adjusted *p*-value threshold of 0.05 (Benjamini-Hochberg method) to ascertain significance.

### Reporter Gene Assays

HEK293 cells were cultured in 96-well plates at a density of 7000 cells per well for 24 h. Following this, NFIA siRNA (Dharmacon J-008661-06) was transfected into the cells at a final concentration of 100 nM using Dharmafect transfection reagent (0.35 μl/well). After 24 h of siRNA silencing, the culture medium was replaced with fresh medium, and the cells were transfected with 50 ng of the SOX2 plasmid or an empty vector (pTO-SH-GW-FRT), along with 47.5 ng of a reporter construct. The reporter construct contained 6× SOX2 binding sites (TFBS: 6× CTTTGT), a minimal promoter, and a firefly luciferase reporter. Additionally, cells were transfected with 2.5 ng of a Renilla-luciferase construct. After another 24 h, the cells were collected, and the firefly luciferase and Renilla luciferase signals were detected using a Dual-GLO luciferase assay system (Promega). Firefly luciferase signals were normalized to Renilla luciferase signals, and the analysis was performed in triplicate.

### Affinity Purification and Proximity Labeling

Approximately 1 × 10^8^ Flp-In T-REx 293 cells stably expressing MAC3N-tagged NFIs were induced with 2 μg/ml tetracycline (AP-MS and BioID) for 24 h and 50 μM biotin added to BioID plates 16 h before the harvesting. The cells were washed and harvested with Harvesting Buffer (1 mM EDTA in 1× PBS), pelleted using centrifugation, snap frozen in liquid nitrogen, and stored at −80 °C. The samples were then suspended in 3 ml of lysis buffer (50 mM HEPES pH 8.0, 5 mM EDTA, 150 mM NaCl, 50 mM NaF, 0.5% NP40, 1.5 mM Na_3_VO_4_, 1 mM PMSF, 1× protease inhibitor cocktail, Sigma) on ice.

BioID lysis buffer was completed with 0.1% SDS and 80 U/ml benzonase nuclease (Santa Cruz Biotechnology), and lysis was followed by incubation on ice for 15 min and three cycles of sonication (3 min) and incubation (5 min) on ice.

All samples were cleared by centrifugation, and the supernatants were poured into microspin columns (Bio–Rad) that were preloaded with 250 μl of (50% slurry) Strep-Tactin beads (IBA GmbH) and allowed to drain under gravity. The beads were washed 3 times with 1 ml lysis buffer and then 4 times with 1 ml HENN buffer. The purified proteins were eluted from the beads with 700 μl of wash buffer containing 0.5 mM biotin. To reduce and alkylate the cysteine bonds, the proteins were treated with a final concentration of 5 mM TCEP (tris(2-carboxyethyl) phosphine) 20 min at 37 °C by shaking and 10 mM IAA (iodoacetamide) for 20 min in dark. Finally, the proteins were digested into tryptic peptides by incubation with 1 μg sequencing grade trypsin (Promega) overnight at 37 °C by shaking. The digested peptides were purified using C-18 microspin columns (The Nest Group Inc.) as instructed by the manufacturer. For the mass spectrometry analysis, the vacuum-dried samples were dissolved in buffer A (1% acetonitrile and 0.1% trifluoroacetic acid in MS-grade water).

### Liquid Chromatography–Mass Spectrometry

The Evosep One liquid chromatography system was utilized to analyze desalted peptide samples. It was coupled to a hybrid trapped ion mobility quadrupole TOF mass spectrometer (Bruker timsTOF Pro 2) via a CaptiveSpray nano-electrospray ion source. Peptides were separated using an 8 cm × 150 μm column packed with 1.5 μm C18 beads (EV1109, Evosep) utilizing the 60-SPD (sample-per-day) method with a 21-min gradient time. Mobile phases A and B were prepared as 0.1% formic acid in water and 0.1% formic acid in acetonitrile, respectively. For all AP and BioID samples, mass spectrometry (MS) analysis was performed in positive-ion mode using a data-dependent acquisition (DDA) strategy in PASEF (Parallel Accumulation Serial Fragmentation) mode, employing the DDA-PASEF-short_gradient_0.5s-cycletime method.

The raw data files were processed utilizing FragPipe v21.1 with MSFragger-4.0 (38) using human protein fasta files containing 40,954 entries (20,477 decoys: 50.0%) from the UniProtKB database (downloaded on January 5, 2024). Carbamidomethylation of cysteine residues was used as static modification while amino-terminal acetylation and oxidation of methionine were used as the dynamic modification. Biotinylation of lysine and N-termini were set as variable modifications. Trypsin was selected as the enzyme with allowance for up to two missed cleavages. The instrument and label-free quantification parameters remained at their default settings. Lastly, the results output consisted of PSM values derived from peptides with a false discovery rate (FDR) below 0.01 as generated by Philosopher.

### Identification of High-Confidence Protein-Protein Interactions

In-house python script, incorporated with Significance Analysis of INTeractome (SAINT) -express version 3.6.3 (39) was used as a statistical approach for identification of specific high-confidence interactions from BioID and AP-MS data. High-confidence interactions (HCIs) were defined by an estimated protein-level Bayesian FDR (BFDR) of ≤0.01 (for BioID) or 0.05 (for APMS). In BioID data, interactions that passed the filter with any bait were rescued for other baits as well. Furthermore, we used our own in-house contaminant GFP library and CRAPome database (version 2.0) (40). Preys detected in over 20% of the GFP library or CRAPome samples were deemed contaminants and removed unless their spectral counts in the sample runs were over 3 times higher than the library average. Preys with an average spectral count of less than three were also removed.

### Data Analysis and Visualization

Bulk tissue expression data of NFIs were obtained from Genotype-Tissue Expression (GTEx) Portal with dbGaP accession number phs000424.vN.pNon April 5, 2024. Enriched Gene Ontology terms (BP, CC, MF, KEGG Pathway) for all NFI interactors were obtained from DAVID Bioinformatics Resources (https://david.ncifcrf.gov/summary.jsp) (41). All the terms with the corresponding *p*-values and FDR are listed in supplemental Dataset S2.

Correlation for biological and technical replicates was analyzed using spectral count values of either biological or technical replicates. Pearson’s correlation coefficient values were calculated for each pair of replicates with the pearsonr method from the Python scipy.stats package (SciPy, version 1.71). Plots for the results were generated with the Python plotly package, RAWGraphs (42), and SRplot webtool (43).

Identified HCIs were compared with known interactors from public interaction databases IntAct (44). CORUM database was used for the analysis of protein complexes in HCI lists (45). NFIs proteome and targetome data were analyzed with the online tool Toppcluster (http://toppcluster.cchmc.org) to generate enrichment networks between the GO terms and pathways and the *p*-value cutoff was set at 0.05. Then networks were imported and edited on Cytoscape software platform (version 3.10.1) to visualize interaction networks of NFIs (46).

SWI/SNF and Mediator complex protein analysis of the 3h and 16h BioID data was performed using ProHits-viz.’s dot plot generation tool (https://prohits-viz.lunenfeld.ca/Dotplot/) (47). Filtered SAINT-interaction files were used as the input. Apart from default settings, the Abundance column was selected as AvgSpec, and the score column was set to BFDR. Filtering cutoff values were removed as filtered SAINT-interaction data were used as input.

### Structural Model Prediction and Analysis

Structural models for NFIA, NFIB, NFIC, and NFIX homodimers in complex with DNA were predicted using AlphaFold3 (48). Initial predictions to confirm binding site specificity were carried out with longer sequences of DNA from ChIP-Seq results (Full length canonical NFI isoforms and 200 nt ChIP-Seq reads) were used, before a final prediction with trimmed DNA sequences. Protein sequences used for each protein were trimmed based on initial predictions to include only the DNA-binding domain and additional structured regions (supplemental Table S1). Interface analysis was performed using the “interfaces” routine in ChimeraX (49), using a probe radius of 1.4 Å, area cutoff of 300 Å^2^, and interface residue area cutoff of 15 Å^2^. Predicted alignment error plots were generated using PAE-viewer (50).

## Results

### General Overview and Validation of the Strategy Used for Global Mapping of Molecular Interactions Involving NFI Family Members

**Fig. 1:**
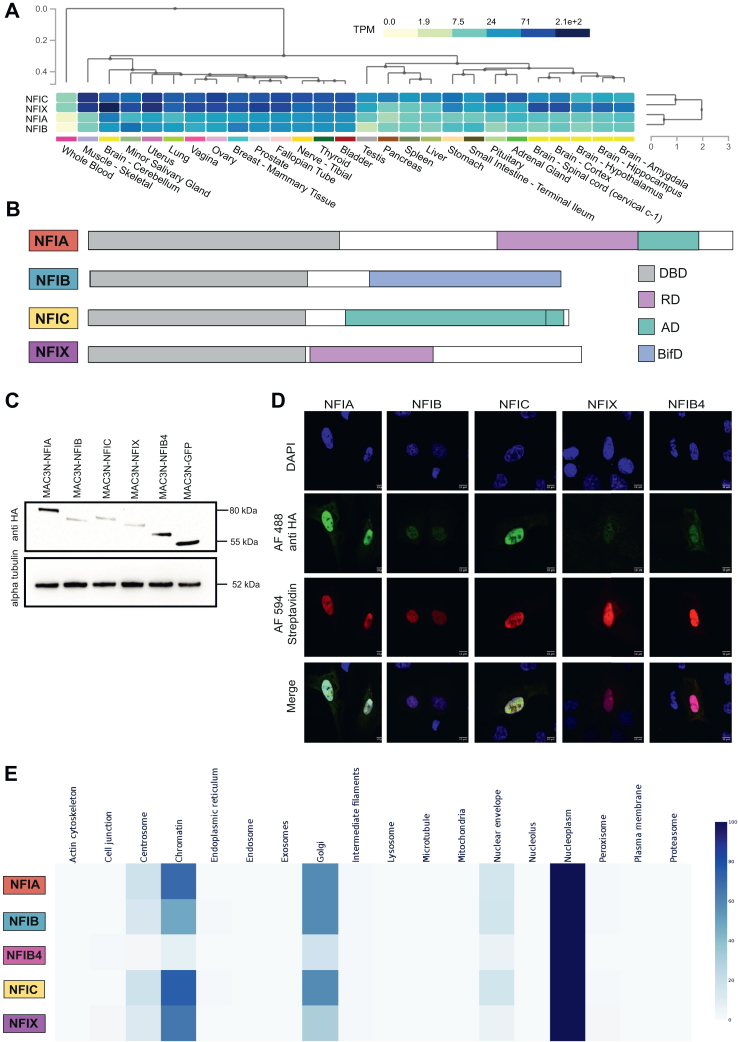
**Analysis of NFI protein expression.***A*, GTex multi-gene tissue expression heatmap visualization for NFI genes. NFIs and tissues are clustered using hierarchical clustering. The expression level of the gene per tissue is color-coded according to the linear count of TPM (transcript per million) as shown with the legend bar. *B*, schematic representation of NFI transcription factors. Each NFI isoform in this study is represented by a box proportional to the size of the open reading frame. The highly conserved N-terminal DNA binding and Dimerization (DBD) domain is indicated as *gray*. In the C-terminal Transcription Activation/Repression domain, the predicted Activation Domain (AD) (*green*), Repression Domain (RD) (*pink*) and Bifunctional Domain (BifD) (*blue*) are indicated in the figure. NFI proteins are color-coded as *NFIA-red*, *NFIB-blue*, *NFIC-yellow*, and *NFIX-purple* and this color-coding applied in all figures. *C*, detection of expression of MAC3-N-tagged fusion proteins by Western Blot analysis. BioID lysates of MAC3-tagged NFIs and GFP detected by anti-HA primary antibody. Alpha tubulin on the membrane is also detected by its antibody as a loading control. *D*, subcellular localization of MAC3-N tagged NFIs in U2OS cells. Fusion proteins visualized by immunofluorescence staining using anti-HA primary antibody and Alexa Fluor 488 as a secondary antibody (*green*), biotinylated proteins were detected with Streptavidin, Alexa Fluor 594 conjugate (*red*), and DNA with DAPI (*blue*). Scale bar, 10 μm. *E*, MS microscopy analysis of interacting proteins revealed by BioID experiments of each MAC3-NFI bait. Most of the NFI interactors are localized in the nucleus, similar to bait protein localization. The color assigned to each of the localizations is based on the annotation score of the MS-microscopy system. The color scale is shown at the right of the panel.

To identify the interaction network(s) of NFI TFs, involving both protein partners and, we designed a robust experimental workflow that integrates the use of interactome-based experiments (BioID and AP-MS), ChIP-Seq, and robust data analysis (supplemental Fig. S1). We enhanced our ability to identify HCIs by using a recently developed MAC3-tag, which comprises of two Strep-Tag II sequences, followed by a HA-tag, and the UltraID enzyme, with flexible linker sequences in between. This design allows for affinity purification, proximity labeling, and ChIP-Seq analyses with a single construct (31, 51, 52, 53, 54). The full-length NFI clones were obtained from the Human ORFeome collection (55), and augmented by the inclusion of NFIB4 as an understudied isoform of interest (supplemental Fig. S1). The expression patterns of NFI family members (NFIA, NFIB, NFIC, and NFIX) across different tissues are shown, demonstrating distinct tissue-specific expression profiles, with NFIA and NFIB showing elevated expression in neural tissues, while other isoforms exhibit varying levels across different tissues (Fig. 1*A*). To ensure consistent protein expression across the cell population, we used the tetracycline-inducible Flp-In T-REx 293 cell lines expressing MAC3-tagged NFIs (supplemental Fig. S1) that achieve expression levels comparable to endogenous proteins (56), which, in turn, ensures physiologically-relevant readout of HCIs and genomic binding sites. Since NFIs’ transactivation/repression domain is located on the C-terminal part of the proteins (Fig. 1*B*), N-terminal tagging was used to avoid disruption of the interactions.

Prior to conducting in-depth interactome and ChIP-Seq analysis, we confirmed the MAC3-tagged NFIs expression and/or subcellular localization. Western blotting confirmed the expression of the NFI proteins, with NFIA and NFIB4 displaying particularly robust expression signals (Fig. 1*C*). Immunofluorescence microscopy revealed predominantly nuclear localization for all the NFI proteins, consistent with their anticipated roles in gene regulation (Fig. 1*D*). Interestingly, the NFIB4 isoform, despite lacking a canonical DNA-binding domain and therefore one of the two NLS sequences, also displayed primarily nuclear localization (Fig. 1*D*). To further investigate the correlation between NFI construct expression and subcellular localization, we conducted MS microscopy analysis of interacting proteins identified through BioID experiments for each MAC3-NFI bait (Fig. 1*E*). Using the MS microscopy platform, we compared the cellular localization of proteins from the BioID interaction list against our reference molecular context proteome map (57). This analysis confirmed previously known localization profiles of NFI TFs (58) and their roles in transcriptional regulation and chromatin organization (6, 18, 59). Notably, despite lacking the DBD, the NFIB4 isoform was also found to primarily localize to the nucleus (Fig. 1*E*). These results validate our ability to identify key HCIs and demonstrate the robustness of our workflow for mapping NFI TF interactomes.

### Time-dependent Analysis of NFI Protein Interactions Using the UltraID Labeling

**Fig. 2:**
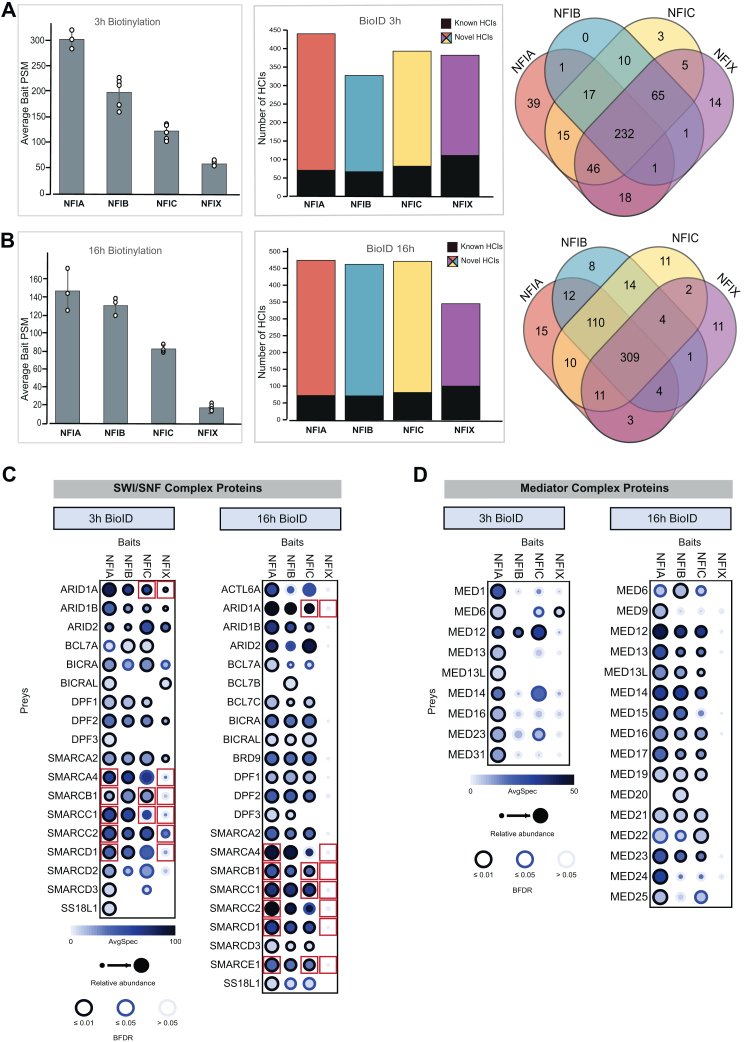
**NFI proximal proteins were revealed by BioID experiments performed using two different biotinylation time.***A*, average number of NFI bait PSM values obtained from BioID replicates with 3 h biotinylation. The number of known and novel high-confidence protein interactors (HCIs) for each NFI protein is shown, along with a comparison of HCI lists using a Venn diagram. *B*, average number of NFI bait PSM values obtained from BioID replicates with 16-hour biotinylation. The number of known and novel HCIs for each NFI protein is displayed, with a comparison of HCI lists using a Venn diagram. *C*, comparison of SWI/SNF complex proteins identified in the 3-hour and 16-hour BioID HCI lists. The ProHits-viz web tool was used to generate a dot plot visualization, where prey average spectral values are represented by color intensity, relative abundance across baits is indicated by dot size, and prey confidence is shown by BFDR values. SWI/SNF complex protein interactions detected in AP-MS experiments are highlighted with *red squares*. *D*, comparison of Mediator complex proteins identified in the 3-hour and 16-hour BioID HCI lists. Dot plots were generated using the ProHits-viz web tool, with prey average spectral values represented by color intensity, relative abundance across baits by dot size, and prey confidence by BFDR values.

Given that gene expression regulation is a dynamic process (60), we wanted to examine the time dependence of NFI TF interactomes using UltraID proximity labeling experiments conducted over 3 and 16 h of biotinylation. Quantitative analysis of the bait abundance was performed using peptide-spectrum match (PSM) values. We observed that NFIA exhibits the highest average bait PSM values among the analyzed NFI members (303.7 and 148 for 3h and 16h, respectively; Fig. 2, *A* and *B*). The NFIB displayed the second-highest average bait PSM (199.4 (3h), 131.3 (16h)), followed by NFIC (123.2 (3h), 83 (16h)), and NFIX (61 (3h), 17.2 (16h)) (Fig. 2, *A* and *B*). Furthermore, average bait PSM counts for all NFI proteins decreased with longer (16h) biotinylation. This trend suggests that the pool of biotinylated proteins expands over time due to prolonged NFI activity, resulting in a larger and more diverse set of interactors captured by the bait proteins at the longer time point (Fig. 2, *A* and *B*). According to the UltraID proximity-labeling results, shorter biotinylation captured a core set of interactions, with both known and novel HCIs (71/369 HCIs for NFIA, 67/260 HCIs for NFIB, 82/311 HCIs for NFIC, 111/271 HCIs for NFIX) (Fig. 2*A* and Table 1). Extending the biotinylation period to 16 h revealed an expanded interaction network (72/402 HCIs for NFIA, 71/391 HCIs for NFIB, 81/390 HCIs for NFIC, 100/245 HCIs for NFIX), suggesting that NFI proteins engage with a broader array of cellular components over time (Fig. 2*B* and Table 1).

**Table 1:** Number of known and novel interactors identified with UltraID experiments

POI	3h biotinylation			16h biotinylation		
	Known HCIs	Novel HCIs	Total	Known HCIs	Novel HCIs	Total
NFIA	71	369	440	72	402	474
NFIB	67	260	327	71	391	462
NFIC	82	311	393	81	390	471
NFIX	111	271	382	100	245	345

When comparing the HCIs identified for individual NFIs with those of others, we observed that a large number of HCIs is shared among all the family members (232 out of 467 total HCIs at 3h; 309 out of 525 at 16h). On the other hand, NFIA, NFIB, NFIC, and NFIX exhibited different patterns of shared and unique HCIs that varied with time in different ways. For example, we did not detect any NFIB-specific HCIs at 3h, whereas we identified eight NFIB-specific HCIs at 16h, suggesting an increase in NFIB-specific function over time. This trend was reversed for NFIA, with 39 NFIA-specific HCIs at 3h and 15 at 16h, suggesting time-dependent decrease in NFIA-specific function. While the mechanisms behind these differences remain unclear, these findings suggest that, despite significant redundancy, each NFI family member exhibits nonredundant roles as reflected in their unique interactomes. Furthermore, this study underscores the time-dependent nature of these interactions.

### Proximity-Labeling with UltraID Reveals NFI Associations with Key Chromatin and Transcriptional Regulators

Our detailed analysis of the NFI interaction networks highlighted significant associations with critical components of chromatin remodeling and transcription regulation complexes, notably SWI/SNF and Mediator complexes, the key players in transcriptional regulation (61, 62). As illustrated in Figure 2, *C* and *D*, NFI TFs exhibited a wide range of interactions with components of both SWI/SNF and Mediator complexes. In both instances, patterns of interactions differ between different NFIs and different subunits, and between the 3h and 16h biotinylation times (Fig. 2, *C* and *D*). In general, 16h time-point captured an increased number of interactions. For example, at 16h time-point proximity-labeling detects 25 out of 29 SWI/SNF subunit proteins as HCIs for NFIB, 24/29 subunits for NFIA, and 23/29 subunits for NFIC (Fig. 2*C*); however, interactions with NFIX were not as prominent. NFIX interacts with a more diverse set of proteins over longer biotinylation times, yielding lower PSM values for bait and prey proteins. As we applied strict filtering parameters for all NFI members, some of the NFIX interacting proteins were filtered out, leading to the less prominent apparent interactome (Fig. 2*C*). We also identified several SWI/SNF complex proteins by AP-MS, a technique that captures only stable protein-protein interactions (supplemental Fig. S2, *A* and *B*, supplemental Dataset S1). The AP-MS showed that SMARCB1, SMARCC1, and SMARCE1 form stable interactions with NFIA, NFIC, and NFIX, SMARCA4, SMARCC2, and SMARCD1 bind NFIA and NFIX (Fig. 2*C* and supplemental Fig. S2*B*). Although NFIA’s interaction with the SWI/SNF complex via ARID1A/B subunits has been previously reported (63) and also observed in our UltraID data, ARID1A co-precipitated only with NFIC and NFIX (Fig. 2*C* and supplemental Fig. S2*B*). To validate the interactions detected for the NFI family members with the SWI/SNF complex members, we performed pull-down and dotblot experiments with four NFI members and 16 SWI/SNF proteins. We could validate all tested 16/16 interactions for NFIA, 15/16 for NFIB, 11/16 for NFIC, and 12/16 for NFIX (supplemental Fig. S3 and supplemental Dataset S11). The total validation percentage was greater than 84% (54 out of 64). Taken together, the NFI TFs engage with the majority of subunits of the SWI/SNF complex, indicative of their close functional relationship.

Using the MAC-tag approach, we have demonstrated that it is possible to determine interaction distances for stable complexes by comparing their Bait Normalized PSM values from AP-MS and BioID experiments (57). We also tested this with NFIA (supplemental Fig. S2*C*) and found that NFIA forms more stable complexes with other NFIs than with transcriptional regulators, such as other transcription factors and chromatin remodelers. While interaction distances are highly similar among transcriptional regulators, SSBP3 is notably more distantly positioned (supplemental Fig. S2*C*).

As mentioned, in addition to SWI/SNF, we also identified several Mediator complex proteins in the proximity-labeling datasets (Fig. 2*D*). Although interactions between Mediator complex subunits at the 3h time-point were primarily centered on NFIA, at 16h NFIA, NFIB and NFIC data indicate engagement with all 16 Mediator subunits we identified (Fig. 2*D*). As noted, NFIX interactions were weak or absent (Fig. 2*D*). In terms of previous data related to NFI-Mediator interactions, Nfia and Nfib were found to interact with mediator complex proteins in mouse neural stem cells (64). Our data corroborates these findings in human cells and additionally uncovers NFIC interaction with MED proteins. However, our AP-MS analysis did not detect any stable interactions between the NFI partners and the components of the Mediator complex (supplemental Dataset S1). These results suggest that interactions between NFIs and the components of the Mediator complex proteins are highly transient and/or proximal.

Collectively, these studies provide evidence for the dynamic nature of NFI-mediated regulation of gene expression. Additionally, these processes are likely primarily driven by direct interactions with the SWI/SNF complex and transient interfacing with the Mediator complex, which in turn drives temporal modulation of chromatin accessibility and direct involvement in the transcriptional machinery.

### Interaction Network of NFI Transcription Factors

**Fig. 3:**
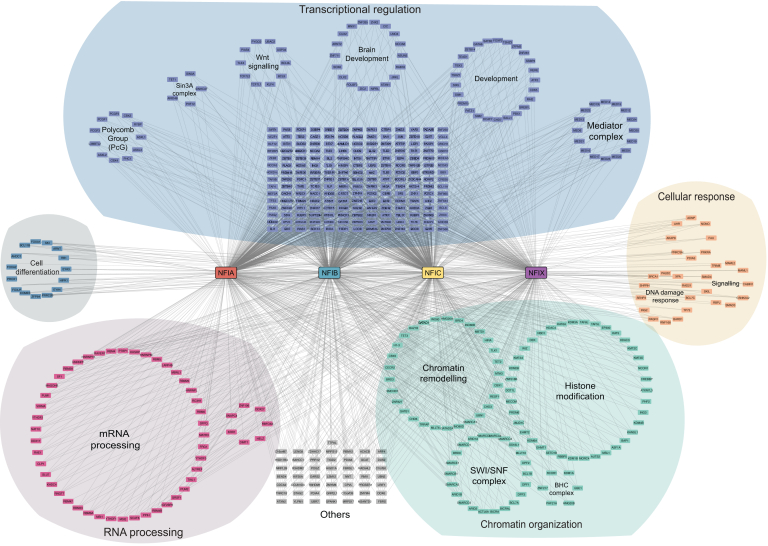
**Comprehensive interactome of NFI transcription factors.** This figure depicts the extensive network of protein interactions associated with NFI transcription factors. Each node represents a distinct protein, and lines denote interactions between them. Proteins are color-coded and grouped by their primary roles in biological processes, including transcriptional regulation, chromatin organization, RNA processing, cell differentiation, and cellular response. Specific functions or complexes within these categories are highlighted as subgroups, enhancing visualization of the complex interplay and regulatory mechanisms mediated by NFI transcription factors in cellular physiology.

Since we were able to detect more complete and in-depth interactomes for the NFI family members with 16h proximity labeling, we used these datasets as the foundation of the comprehensive NFIs interactomes. Overall, we obtained 17,279 hits (unfiltered data) for all four NFI members (data not shown), and, after data filtering, a total of 1752 HCIs were identified (supplemental Dataset S1). Among these, 309 HCIs are shared by all NFI members we profiled, suggesting functional redundancy in pathways and parts of networks involving these HCIs, most notably BCOR, KMT2D, MGA, CREBBP, ARID1A, TLE3, CIC, NCOR1, NCOR2, and PRR12. This is in agreement with the existing literature and our prior interactome studies (25, 65, 66). In addition to known interactors, we identified novel interactors shared by all NFI members with high spectral count values such as JMJD1C, ZFHX4, SMARCC2, SMARCA4, HNRNPM, and ARID2. Importantly, we also identified key points of difference. For example, only NFIA displayed interaction with KLF4, MAML2, and ZDHHC17, NFIB interacted uniquely with MED20, BCL7B and CDK5, NFIC displayed specific interactions with FOXA2 and E2F7, and NFIX-specific HCIs included KDM4B and KCTD15 (Figs. 2*B* and 3 and supplemental Dataset S1). Additionally, NFIB4 also specifically interacted with mRNA-related proteins PTBP2, QKI, TIA1, ESRP2, RBPMS, and CPEB4 emerging as unique hits (supplemental Dataset S1); however, the functional relevance of these interactions remains to be validated. Taken together, these studies showcase both the shared and distinct features of the NFI TF interactomes and support the hypothesis that different NFI TFs use distinct interaction partners to exert specific, context-dependent functions.

To map a more complete interaction network of NFI TFs beyond SWI/SNF and the Mediator, we analyzed our 16h proximity-labeling data, given its more comprehensive coverage of the interactome (supplemental Dataset S1). We mapped all HCIs to the four NFI TFs included in our UltraID analysis, resulting in a comprehensive interaction network of NFI TFs displaying all interacting proteins classified according to their primary roles (Fig. 3). This network demonstrates that NFI TFs play different roles in a wide range of processes by interfacing with other proteins involved in transcriptional regulation, chromatin organization, mRNA regulation, and cellular signaling pathways (Fig. 3).

The majority of the NFI-associated proteins are integral to transcriptional regulation and chromatin organization, underscoring NFIs’ essential roles in regulating gene expression. The network reveals that NFI TFs interact with key components involved in chromatin remodeling and histone modification suggesting that NFI TFs are directly involved in epigenetic regulation. These interactors include the SWI/SNF complex, Polycomb-group proteins, and elements of the Sin3A complex, consistent with their roles in gene expression modulation via chromatin structure alterations. Interestingly, we identified 45 mRNA processing-related proteins in the NFI interactome (Fig. 3), suggesting the possibility that NFIs play a role in mRNA splicing and stability. This, for the first time, hints that NFIs may also impact post-transcriptional gene regulation. However, whether these interactions are due to colocalization, or coincidental transcription/splicing remains to be investigated.

In addition to these networks, NFI TFs also showed novel connections to proteins involved in cell differentiation and developmental processes, such as, GATA6, ATXN1, FOXP2, and TBX3. To further dissect pathways and cellular processes impacted by NFI TFs, and provide a more granular view, we performed a GO Biological Process enrichment analysis for HCIs of NFIA, NFIB, NFIC, and NFIX. This analysis uncovered positive and negative regulation of transcription and chromatin organization-related processes as highly enriched (supplemental Dataset S2 and supplemental Fig. S4*A*). For example, transcription factor complexes, the Mediator complex, and SWI/SNF complex were found to be highly enriched (supplemental Fig. S5*A*). Additionally, GO Molecular Function (GO-MF) analysis showed mainly transcription factor activity, and DNA and chromatin binding molecular functions of NFI interactors (supplemental Dataset S2 and supplemental Fig. S5*B*). Enrichment analyses also demonstrated a notable spatial co-localization of the HCIs with NFIs, further supporting our conclusions that these interactions are functionally relevant (supplemental Dataset S2 and supplemental Fig. S5). We also performed the KEGG pathway enrichment analysis for NFI HCIs. Pathways that exhibited significant enrichment (Benjamini <0.001) include “Lysine degradation”, “Notch Signaling Pathway” and “Wnt signaling pathways”, thyroid hormone signaling, and thermogenesis, indicating a broad impact of NFIs on cellular signaling. Incidentally, lysine degradation and thermogenesis pathways generally comprised proteins involved in histone modification, mostly SWI/SNF complex proteins. Additionally, NFI interactors enriched by SWI/SNF complex members in “Hepatocellular carcinoma” and “Transcriptional misregulation in cancer”, point to a potential role of NFI proteins in oncogenesis and cancer progression (supplemental Dataset S2 and supplemental Fig. S4*B*).

Collectively, the interaction network highlights the complex roles of NFI TFs, emphasizing their importance in transcriptional regulation, cellular development, and response to environmental signals. Furthermore, the comprehensive pathway analysis underscores the role of NFI TFs in health and disease, offering insights into their biological roles and therapeutic potential.

### Genome-Wide Analysis of NFI Occupancy and Enrichment of Potential Targets

**Fig. 4:**
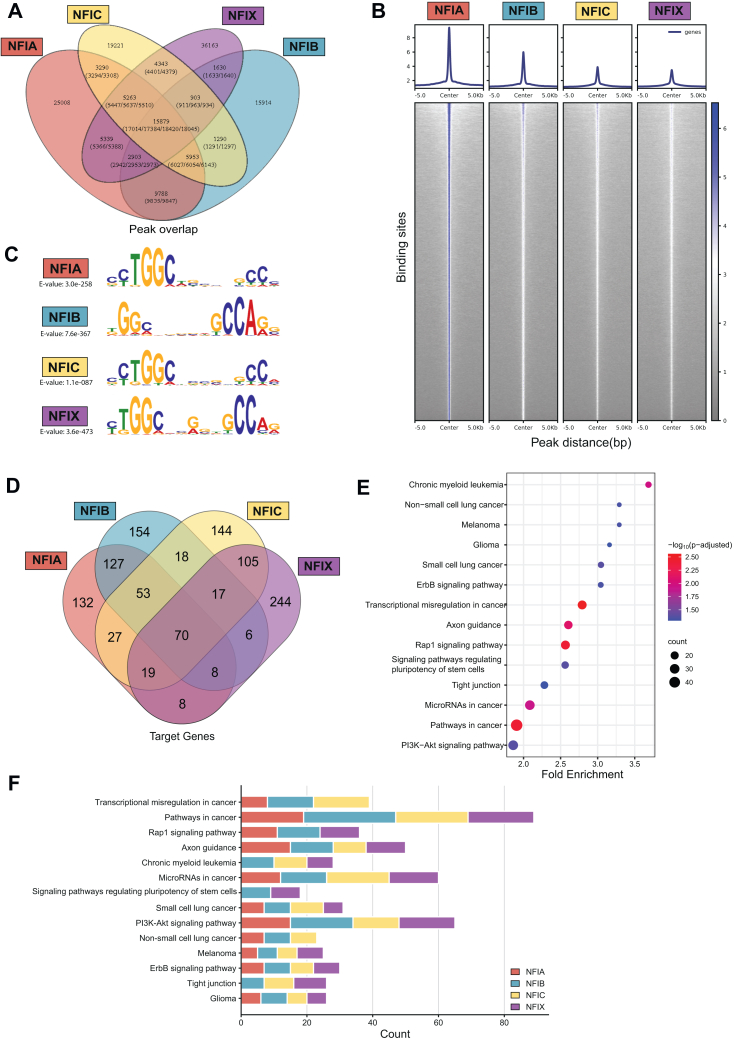
**Chromatin immunoprecipitation sequencing analysis of NFI target genes.***A*, Venn diagram showing overlapping peaks shared between NFIA, NFIB, NFIC, and NFIX. *B*, Heatmap representation of NFIs binding intensity based on ChIP-Seq signals in 293T cells. NFIA is used as the reference, and the heatmaps for the other NFIs are adjusted relative to this reference panel. Signals within 5 kb around the center of binding peaks are displayed. The average signal of each NFI member’s binding is shown in the upper part. *C*, most enriched motifs found as a target of each NFI. *p* values are shown on the *left*. *D*, Venn diagram showing overlapping potential target genes of NFIs, focusing on the top 500 peak sequences. *E*, the pathway enrichment analysis of all NFIs ChIP-Seq targeted genes. Pathways are considered as enriched according to Benjamini values (adjusted *p* value) less than 0.05. Dot size represents the number of target genes involved in each pathway and is shown in the *left panel* together with a color scale bar of -log10(*p*-adjusted). *F*, stacked bar graph showing gene counts of enriched pathways for each NFI member.

To characterize the genome-wide binding occupancy of the NFI family members, we performed ChIP-Seq analyses for all four NFI isoforms. This investigation revealed an excess of 55,000 binding peaks for each isoform, underscoring the pronounced DNA-binding activity of the NFI family. Notably, within these transcription factor binding sites (TFBS), 15,879 peaks exhibited overlap among the NFI members (Fig. 4, *A* and *B*). Some of the ChIP-Seq targets include several known targets of NFI proteins, such as *CDON*, *Ezh2*, and *Hes1*, which have been previously identified in various cellular contexts. We found that the *CDON* regulatory region was occupied by NFIA and NFIB (supplemental Dataset S3), aligning with findings that NFIB represses *CDON* in neuroblastoma cells (67). In neural stem cells, NFIs suppress self-renewal associated genes such as *Ezh2* and *Hes1* (68, 69), and we detected *Ezh2* as the target of NFIC and *Hes1* as a target of all four NFIs (supplemental Dataset S3). The consistency between published studies and our data strengthens confidence in our ChIP-Seq analysis.

Thus, we analyzed our data further and calculated the most enriched DNA motif targeted by each NFI, along with their statistical significance (*p*-values) (Fig. 4*C*), and observed that they correspond to the known DNA-binding preferences of the NFI family. To investigate the structural basis for target recognition of NFIs, we carried out structural predictions using AlphaFold3 (48) (supplemental Fig. S6). We used sequence hits, which included the most enriched motifs (Fig. 4*C*), from ChIP-Seq results as binding partners for homodimeric NFI sequences and found that they converged on single unique sites within each DNA sequence (supplemental Fig. S6, *A* and *B*). We noted that although they bind slightly different DNA sequences, the NFI TFs engage target DNAs via four completely conserved regions (loop, helix1, helix2, and segment; supplemental Fig. S6*C*). These predictions reveal conserved structural elements in NFIs that are crucial for target recognition. Despite their structural similarity, these regions can bind to a diverse range of DNA motifs. The well-known motif for NFIs is TTGGC(N5)GCCAA, where N typically represents five variable bases (the degenerate five nucleotide spacer region) between the palindromic sequences (70). However, our analysis shows that NFIs can also bind motifs with spacer regions of differing lengths, as observed in our ChIP-Seq data (supplemental Fig. S6*C* and supplemental Table S1). This observation aligns with previous findings, which demonstrated that variations in spacer length can significantly reduce DNA-binding efficiency, with some changes leading to a complete loss of binding (70, 71). These findings suggest that variations in spacer length likely modulate the spacing and orientation of the conserved structural domains necessary for DNA recognition. This flexibility in motif recognition indicates that, while NFIs predominantly interact with their canonical consensus sequence, they can adjust to variations in spacer length, potentially extending their regulatory influence across different genomic regions.

We further analyzed the overlap among potential target genes, focusing on the top 500 peak sequences selected based on peak significance. We found 70 potential target genes shared by all four NFI members (Fig. 4*D*). Additionally, each NFI member displayed a unique set of target genes, 132, 154, 144, and 244 unique targets for NFIA, NFIB, NFIC, and NFIX, respectively (Fig. 4*D*). This analysis underscores the complexity of NFI-mediated gene regulation, with each TF contributing both shared and unique regulatory roles.

The KEGG pathway enrichment analysis of ChIP-Seq targets identified several significantly enriched pathways, including “Transcriptional Misregulation in Cancer,” “Pathways in Cancer,” “Rap1 Signaling Pathway,” “Axon Guidance,” and “Chronic Myeloid Leukemia”. Notably, the most enriched pathway is “Pathways in Cancer,” with 47 NFI target genes involved, underscoring the significant role of these transcription factors in cancer biology. Additionally, the “ErbB Signaling Pathway,” “Tight Junction,” “Glioma,” and “Small Cell Lung Cancer” pathways are also prominently represented, further highlighting the broad regulatory impact of NFIA, NFIB, NFIC, and NFIX on several types of cancer (Fig. 4, *E* and *F*). The analysis also revealed that many cancer-related pathways are commonly regulated by multiple NFI proteins (Fig. 4*F*). The only exceptions are “Signaling pathways regulating pluripotency of stem cells”, which is influenced only by NFIB and NFIX (Fig. 4*F*). Building on this, we also performed the GO Biological Process (GO-BP) enrichment analysis revealing enrichment of the following biological processes: “regulation of transcription from RNA polymerase II promoter”, “apoptotic process”, “nervous system development”, and “chromatin remodeling” (supplemental Fig. S7*A*). Comparatively speaking, each member of the NFI family displayed both overlapping regulatory roles (*e.g.* involvement in “positive regulation of transcription from RNA polymerase II promoter” and “negative regulation of transcription, DNA-templated” regulated by all four NFI members) and in a unique set of processes (*e.g.* “male gonad development”, “BMP signaling pathway” and cellular “senescence” found as targets of NFIB, NFIC, and NFIX only) (supplemental Fig. S7*B*).

Overall, ChIP-Seq analyses comprehensively delineated the binding patterns, motif enrichment, gene targeting, and pathways potentially regulated by NFI TFs, providing valuable insights into their genomic and regulatory landscapes. This extensive profiling confirms the critical role of NFIs in regulating key biological pathways and processes across the genome.

### Integrating Interactome and Targetome Analyses Reinforces the Role of NFIs in a Wide Range of Cellular Processes and Pathways

**Fig. 5:**
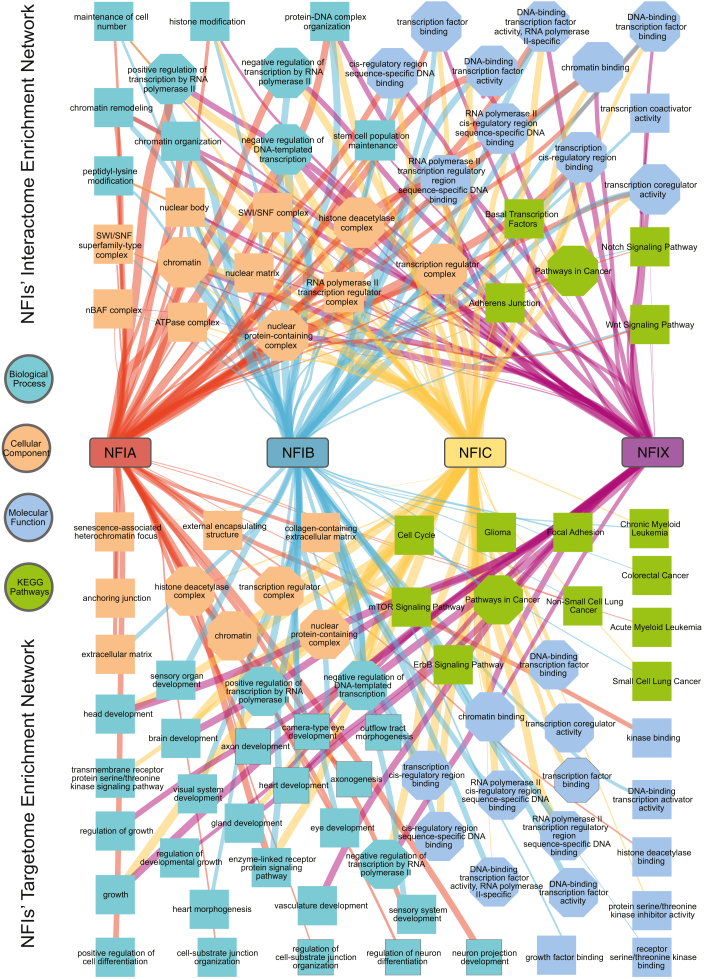
**Gene Ontology (GO) enrichment networks of NFI interaction partners and downstream targets.** All NFIs high- confidence interactor proteins (UniProt IDs) and ChIP-Seq data target genes (human Entrez Gene IDs) were used as input for the ToppCluster. The following databases were selected as “biological process”, “cellular component”, “molecular function” and “pathway”. Networks were imported in Cytoscape 3.10.1 for further modifications. Nodes for the three GO functions and pathways were in different colors and the color key is shown on the *left*. Common terms shared between interactome and targetome enrichment are shown as octagons. Edge thickness is proportional to the number of proteins/genes involved in particular terms and the ratio is 1:10 for KEGG pathways and ChIP-Seq GO terms, and 1:30 for interactome GO terms.

To integrate the results of the interactome and targetome analyses into a unified view of NFIs, we used NFIs’ HCI proteins identified in the BioID experiment and target genes derived from ChIP-Seq datasets to generate a comprehensive overview of the functional categories enriched among the interaction partners and downstream targets of NFI TFs (Fig. 5). Octagonal nodes indicate common terms that are enriched both in the NFI interactome and targetome, highlighting overlap between proteomic and genomic analyses. Among the enriched biological processes, significant terms include “gene expression regulation”, “signal transduction”, and “cell cycle regulation”. These processes underscore the broad regulatory scope of NFI TFs. In terms of cellular components, the enrichment analysis shows a significant presence of NFI-related proteins and target genes in components like “nucleus,” “chromatin,” and “transcription factor complexes.” This localization reflects the fundamental role of NFIs in chromatin dynamics and transcriptional regulation directly at the chromatin. Molecular function analysis emphasizes the role of NFIs in “DNA binding,” “transcription factor activity,” and “protein dimerization activity” (Fig. 5). These molecular functions are crucial for the transcriptional regulation capabilities of NFI proteins, facilitating direct interaction with DNA and other proteins necessary for initiating and regulating transcription. Moreover, NFI proteins are notably involved in “cancer,” “Notch signaling pathway,” and “Wnt signaling pathway.” Collectively, both proteomic and genomic data provide a consistent view of NFI TFs as key regulators of gene expression across the genome, thus impacting a wide range of processes and pathways.

### Detailed Analysis of NFIA Interactome and Its Regulatory Influence on SOX2 Activity and Genomic Binding

**Fig. 6:**
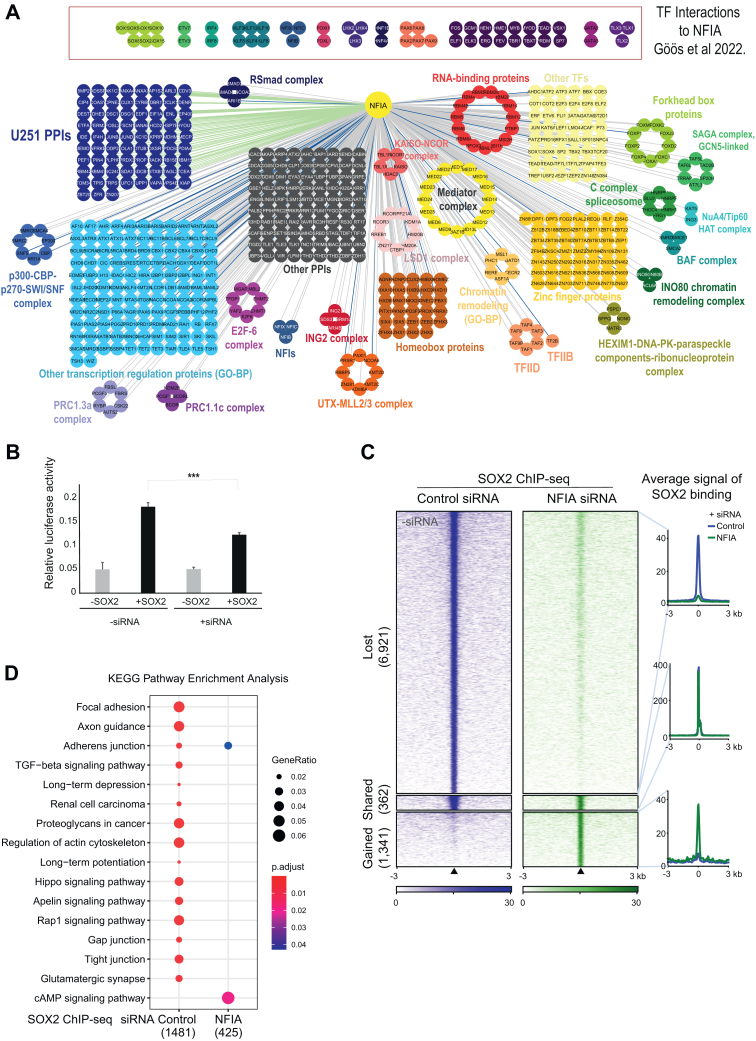
**NFIA Interactome analysis and effect of NFIA silencing on SOX2 activity.***A*, the NFIA protein interactome comprises 602 high-confidence protein-protein interactions. Among these, 421 interactions were identified in the HEK293 model (represented by *gray edges*), 53 interactions were common to both the HEK293 and U251 models (indicated by *blue edges*), and 75 interactions were exclusive to the U251 model (depicted by *green edges*). Additionally, transcription factor (TF) interactions with NFIA, as reported in the recent publication by Göös *et al.* (25), are highlighted in a box above the interaction map. Furthermore, protein complexes and groups, based on CORUM complexes and Biological Process GO terms, are color-coded for clarity. *B*, NFIA was silenced using siRNA transfection, and the activity of SOX2 was investigated through reporter gene analysis both with and without SOX2. In the presence of siRNA, SOX2 activity was significantly reduced (*p* < 0.001). *C*, Heatmap representation of SOX2 binding intensity based on ChIP-Seq signals in 293T cells while treated with siControl and siNFIA, respectively. Signals within 3 kb around the center of binding peaks are displayed in descending order for each SOX2 binding event (lost, shared, and gained upon siNFIA). The plots displaying the average signal of SOX2 binding at each corresponding region are shown on the *right* side of the heatmap. *D*, the pathway enrichment analysis of SOX2 ChIP-Seq targeted genes was conducted upon treatment with siControl or siNFIA. Notably, under the NFIA depletion condition, the pathways enriched were significantly reduced compared to siControl. Interestingly, the cAMP signaling pathway was specifically enriched in siNFIA, while it was not observed in siControl.

To dive deeper into a single NFI and illustrate the ability of our datasets to inform new biological insights, we examined the NFIA interactome in the U251 malignant glioma cell line due to the critical role of NFIs in glioblastoma (18) and compared it to the HEK293 interactome. Analysis of the NFIA protein interactome identified 602 HCIs, with 53 interactions in common between HEK293 and U251 cell lines. These shared HCIs are indicative of shared core mechanisms that exist in HEK293 and U251 cell lines. Interestingly, 421 HCIs were specific to the HEK293 cells, and 75 HCIs were exclusive to the U251 cells (supplemental Datasets S1 and S4). These differences suggest cell-type-specific roles for NFIA. Detailed mapping of these results underscores the highly extensive TF-TF interactions in which NFIA is involved, as well as associations with SWI/SNF complex, BAF and SAGA complexes, INO80 chromatin remodeling complex and others, many of which are connected with regulation of gene expression (Fig. 6*A*).

These results confirm our previous observations that NFIA regulates the function of several TFs (25). To further explore one of these relationships, we focused on SOX2, a TF that was identified as an HCI by us and others (25, 72), as well as designated as an NFIA binder in the String database (https://string-db.org/network/homo_sapiens/NFIA). In reporter assays, siRNA-mediated knockdown of NFIA led to a considerable reduction in SOX2 activity, indicating a strong regulatory relationship between NFIA and SOX2 (Fig. 6*B*). To further examine whether NFIA regulates genome-wide chromatin binding of SOX2, we performed ChIP-Seq in NFIA depleted HEK293 cells. The heatmap representation showed changes in SOX2 binding sites within 3kb around binding peaks, categorized into lost, shared, and gained events post-silencing. This analysis revealed the substantial loss of 6921 SOX2 binding sites, while only 362 sites remained and 1341 were gained (Fig. 6*C*). Consistent with this global shift in the genomic binding profile, depletion of NFIA led to a drastic difference in pathway enrichment for SOX2 target genes. Pathway enrichment analysis of SOX2 target genes post-NFIA silencing identified a specific enrichment of the cAMP signaling pathway highlighting a novel aspect of NFIA’s regulatory impact not observed under control conditions (Fig. 6*D*). NFIA’s functional regulatory effect on SOX2, another TF, suggests that NFIA might contribute to the pathophysiological functions of its interacting TFs on a genome-wide scale.

### Identifying Differential Protein Interactomes of NFIB and NFIB4

**Fig. 7:**
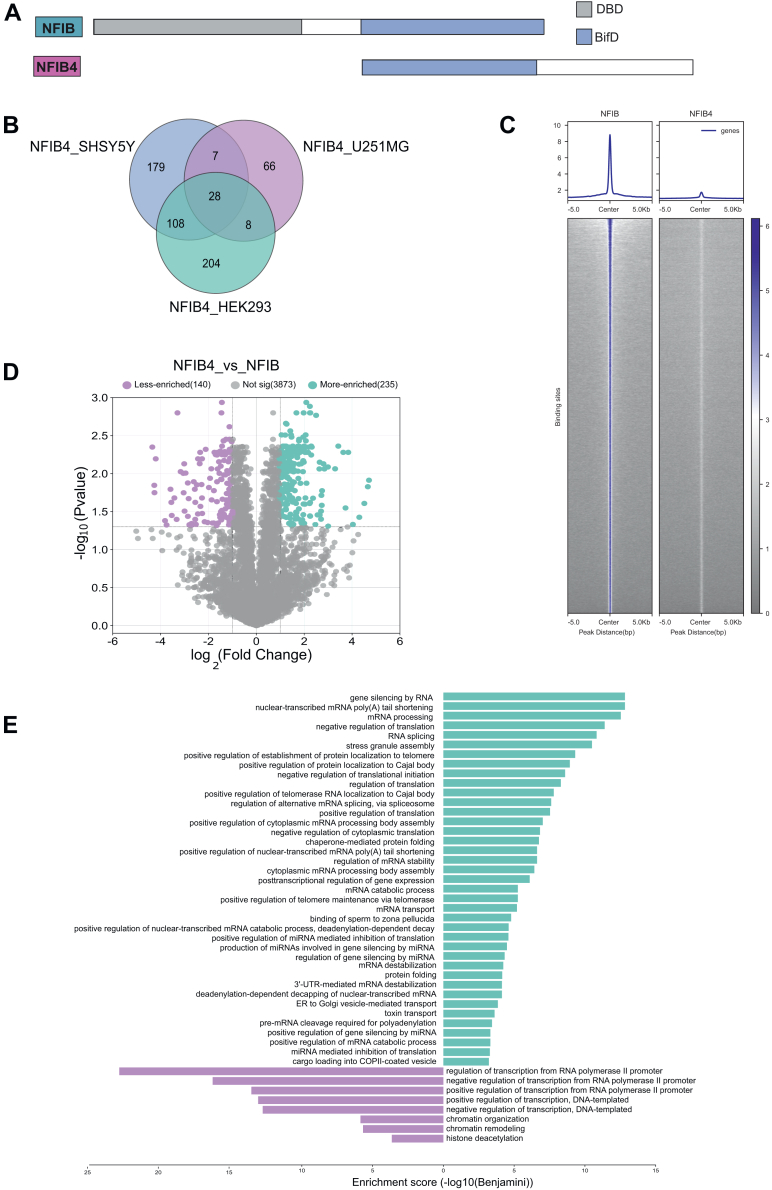
**HCIs Comparison of short isoform NFIB4 and full-length NFIB.***A*, schematic representation of NFIB isoforms. Each NFIB isoform in this study is represented by a box proportional to the size of the open reading frame. The highly conserved N-terminal DNA binding and Dimerization (DBD) domain in full-length isoform, is indicated as *gray*. Identical Bifunctional Domain (BifD) (*blue*) regions are aligned to show sequence similarity and differences in two NFIB isoforms. *B*, number of high-confidence protein interactors of NFIB4 isoforms obtained from three different cell lines (SH-SY5Y, U251-MG, and Flp-in TRex HEK293) and comparison of the HCI lists by Venn diagram. *C*, heatmap representation of full-length NFIB and NFIB4 binding intensity based on ChIP-Seq signals in 293T cells. NFIB is used as the reference, and the heatmap for NFIB4 is adjusted relative to this reference panel. Signals within 5 kb around the center of binding peaks are displayed. While strong NFIB binding signals are observed, NFIB4 binding signals are not detected. *D*, volcano plot of differentially expressed proteins in NFIB4 against NFIB (log2-fold-change threshold = 1, Benjamini corrected *p*-value threshold = 0.5). *Pink dots* show two and more than two fold less-enriched proteins in the NFIB4 list (140 proteins), and *green dots* show two and more than two fold more-enriched proteins in NFIB4 list in comparison with the NFIB list. *E*, GO Biological process (GO-BP) enrichment analysis of differentially expressed proteins in NFIB4 list. *Green bars* show biological processes of upregulated 235 proteins while *red bars* show the biological processes of downregulated 140 proteins in the volcano plot (Benjamini <0.001).

NFIB4 isoform lacks the N-terminal DBD present in other NFI members, as well as the 15-amino acid nuclear localization signal (NLS1) (Fig. 7*A*), suggesting that this isoform may play cytoplasmic roles. However, our subcellular localization analysis revealed that NFIB4 is primarily present in the nucleus (supplemental Fig. S1). To examine this further and compare NFIB and NFIB4, we performed proximity labeling experiments in three cell lines: HEK293, SH-SY5Y neuroblastoma cells, and U251 MG glioblastoma cells. Our analysis revealed 28 HCIs that are shared across all three cell lines, 123 HCIs found in two out of three cell lines, 179 HCIs only in neuroblastoma, and 66 HCIs only in glioblastoma (Fig. 7*B*) (supplemental Dataset S4). These results indicate that in addition to shared HCIs, which are likely representative of core cellular processes, NFIB4 also exhibits cell line-specific interactions that support context-dependent functions.

We employed ChIP-Seq to elucidate the genome-wide binding sites of NFIB4. As anticipated, we did not detect any NFIB4 binding sites (Fig. 7*C*), corroborating the premise that DNA-binding activity is intrinsic to the N-terminal domain of NFIB. This outcome further underscores the specificity and critical role of the NFIB DNA-binding domain in genomic interactions.

The comparative analysis between the short isoform NFIB4 and the full-length NFIB, as presented in the volcano plot, revealed a significant differential abundance of detected proteins. Notably, 140 proteins were found to be less enriched (pink dots) in NFIB4 compared to NFIB (Fig. 7*D*). In contrast, 235 proteins were identified as more enriched (green dots) in NFIB4 (Fig. 7*D*). These data suggest different regulatory roles for NFIB4 and NFIB. The GO-BP enrichment analysis of differentially expressed proteins associated with NFIB4 revealed significant enrichment in proteins involved in “RNA splicing”,“mRNA processing”, and “regulation of translation” (Fig. 7*E*). This implies that, unlike NFIB primarily engages in the regulation of transcription, NFIB4 may be involved in regulating post-transcriptional events. Consistently, NFIB4 did not interact with proteins (red bars) involved in transcription and chromatin organization. This enrichment analysis suggests that NFIB4 modulates gene expression at the mRNA level, distinguishing it from the full-length NFIB's regulatory impact at the DNA level.

## Discussion

Here, we present a comprehensive view of the NFIs’ functional landscape using the combination of interactomics (AP-MS and proximity labeling) and ChIP-Seq. By employing these advanced methodologies, we uncovered novel insights into the dynamics and functional implications of NFIs’ protein-protein interactions and downstream targets. We showed that NFIs predominantly localize to the nucleus, consistent with their role in gene regulation. Notably, even NFIB4, an isoform that lacks a canonical DBD and NLS, was found primarily in the nucleus, which suggests potential novel functions or mechanisms of action for this isoform. Using a time-dependent interactome mapping, we observed that interaction landscape and the effects of NFIs expand over time, suggesting temporal modulation of NFI interactions. We found that transcription factors exhibit slower interaction dynamics compared to protein kinases (52). This slower pace aligns with their role in regulating gene expression over extended periods, orchestrating developmental processes, and maintaining cellular identity. Unlike the rapid signaling events seen in receptor tyrosine kinases, transcription factors like NFIs are involved in sustained gene regulatory activities, which require precise and gradual modulation of their interactomes. The GO enrichment analysis of NFI interactome and targetome revealed the involvement of NFIs in transcriptional regulation, chromatin organization, cellular signaling pathways, and pathways related to cancer. The enrichment of common terms among potential target genes and interaction partners demonstrates a strong correlation between genomics and proteomics data and highlights the interconnected nature of NFI functions at both the genetic and protein levels.

In general, NFIs play a major role in regulating the expression of target genes by cooperating with or antagonizing other TFs, as well as by altering chromatin accessibility. Some examples are NFIB and NFIX’s role in super-enhancer maintenance of hair follicle stem cells (9), NFIB’s prometastatic actions in small cell lung cancer (73), and the recent identification of NFIB as a genome organizer in the prereplication complex (74). Indeed, we found significant NFI association with the SWI/SNF and Mediator complexes which are essential players in transcriptional machinery, and we were able to detect a larger number of complex proteins with increasing biotinylation time. While NFIA and NFIB, together with SOX2 and other TFs, had been identified as interactors of the Mediator complex in a neural stem cell line (64), this study finds that NFIC also interacts with most of the Mediator complex proteins.

In addition to providing global and integrated views of interactome and targetome across the family, our studies also extend the current understanding of individual NFIs. For example, previous work discovered that, during gliogenesis, the cell fate switch between astrocytes and oligodendrocytes involves NFIA interaction with SOX9 and SOX10, SOXE family proteins (27, 28). Here, we find that NFIA knockdown leads to a great reduction in genomic sites occupied by SOX2 indicating that SOXB1 family members may also require NFIs for their regulatory functions. This is aligned with previously reported interactions between NFIB and SOX2 in NS5 neural stem cells (75, 76). Furthermore, the link between NFIs and the SOXB1 family may also be through NFI-mediated upstream regulation of SOX2 expression, as noted in breast cancer cells, dental epithelial stem cells (DESCs), dental pulp stem cells, and bone marrow stem cells (77, 78). Interestingly, NFI motifs are enriched in SOX2-occupied sites in ES cell-derived neural progenitors (79). Therefore, this suggests that SOX2 site binding depends on NFIA across the genome, highlighting the importance of NFIs in maintaining regulatory networks that govern cell fate decisions, stem cell maintenance, and differentiation. As NFIs are expressed later in embryonic development, it remains to be seen if SOX2 cooperates with NFIA, or other NFIs, in the regulation of stem cell maintenance and differentiation.

Importantly, although they possess highly homologous DBDs and bind DNA as either homodimers or heterodimers with similar affinity, we observed that in addition to shared HCIs, all four main family members, NFIA, NFIB, NFIC, and NFIX, engage unique targets as well and exhibit distinct patterns of time-dependence. Therefore, we posit that one of the main drivers of specific activity is the ability of each family member to interact with a unique set of binding partners resulting in distinct functional outcomes. Additionally, we utilized AlphaFold3 to investigate the structural basis of target recognition by NFIs, revealing four conserved binding regions—loop, helix1, helix2, and segment—crucial for DNA interaction. Using sequence hits from ChIP-Seq experiment results, we predicted homodimeric NFI binding to potential targets and demonstrated their homodimer formation and dimeric binding to DNA. Despite the uniformity in these binding domains, NFIs demonstrated remarkable versatility in binding different DNA sequences, suggesting a flexible mechanism that enables the regulation of diverse genetic targets. This is likely further modulated via NFI heterodimer formation, a point that needs further investigation. Taken together, the ability to engage a broad range of binding partners, as well as isoform-specific interactors, and the flexible mechanism of DNA sequence binding elucidates NFIs’ functional redundancy and specificity, and the impact of these TFs on a wide range of cellular processes.

Additionally, NFI functional specificity can arise from a diversity of alternatively spliced isoforms, such as NFIB4. NFIB4 was previously identified as a regulator of megakaryocytes (MK) differentiation (80), although given its lack of the DBD, numerous functional and mechanistic questions about this regulation remained open. We find that NFIB4 predominantly localizes to the nucleus and preferentially engages with proteins involved in mRNA processing and cellular stress responses, distinguishing it from the canonical functions of full-length NFIB. These data provide insight into how NFIB4 and other predicted truncated isoforms may function in gene regulation.

Future research on the NFI family should focus on elucidating the mechanisms underlying their interactions and specific roles in various cellular contexts, including the roles of their alternatively spliced isoforms. Additionally, understanding how NFIs engage with other transcription factors and regulatory complexes over time and in different tissues could reveal more about their context-dependent functions. Comparative studies of the regulatory roles of different NFI isoforms in cancer and developmental diseases might offer insights into potential therapeutic interventions. Moreover, expanding the scope of research to include more cell types and disease states could help identify novel pathways and targets regulated by NFIs, deepening our understanding of their roles in health and disease.

Recent work shows NFIB as well as NFIA can act as master (reprogramming) transcription factors in astrocyte cell differentiation (81, 82). Others have shown that NFIB acts a pioneer factor in DNA replication (74), promotes chromatin accessibility in SCLC (73), and along with NFIX, contribute to the hair follicle niche (9). Moreover, ATAC-seq data suggest similar roles for NFIA and NFIB in the SHH subgroup of medulloblastoma, NFIB, and NFIX in prostate cancer, respectively (83, 84). Here, we provide evidence for extensive interactions with chromatin modeling complex proteins (such as SWI/SNF complex, BAF and SAGA complexes, INO80 chromatin remodeling complex, Fig. 6*A*) and these interactions elucidate mechanisms by which NFI proteins can act as genome organizers in general. Intriguingly, these mechanisms could also explain how NFIA binds to and regulates the activity and DNA binding of another transcription factor, SOX2.

In conclusion, our study elucidated the dynamic and context-dependent nature of NFI protein-protein interactions and provided valuable insights into their functional implications in gene regulation and cellular physiology. Our identification of specific NFI targets further underscores their significant role in modulating gene expression. These findings contribute to our understanding of NFI biology and highlight potential avenues for future research in this field.

## Data Availability

The raw mass spectrometry file names for all samples in this study (a total of 84 runs) are detailed in supplemental Dataset S5. All raw mass spectrometry data have been deposited in the MassIVE repository (massive.ucsd.edu; accession number: MSV000095163). Protein identification files from FragPipe v21.1 with MSFragger-4.0 search are given in supplemental Dataset S6 (HEK-BioID-16h), supplemental Dataset S7 (HEK-BioID-3h), supplemental Dataset S8 (HEK-AP-MS), supplemental Dataset S9 (SH-SY5Y-BioID), supplemental Dataset S10 (U251-BioID) including all individual sample files. All raw ChIP-seq data have been deposited in the European Nucleotide Archive (ENA) under accession ID PRJEB79158.

## Supplemental data

This article contains supplemental data.

## Conflict of interest

The authors declare that they have no conflicts of interest with the contents of this article.

## References

[1] Nagata K., Guggenheimer R.A., Enomoto T., Lichy J.H., Hurwitz J. (1982). Adenovirus DNA replication in vitro: identification of a host factor that stimulates synthesis of the preterminal protein-dCMP complex. Proc. Natl. Acad. Sci. U. S. A..

[2] Fane M., Harris L., Smith A.G., Piper M. (2017). Nuclear factor one transcription factors as epigenetic regulators in cancer. Int. J. Cancer.

[3] Harris L., Zalucki O., Clément O., Fraser J., Matuzelski E., Oishi S. (2018). Neurogenic differentiation by hippocampal neural stem and progenitor cells is biased by NFIX expression. Development.

[4] Piper M., Gronostajski R., Messina G. (2019). Nuclear factor one X in development and disease. Trends Cell Biol..

[5] Chen K.-S., Bridges C.R., Lynton Z., Lim J.W.C., Stringer B.W., Rajagopal R. (2020). Transcription factors NFIA and NFIB induce cellular differentiation in high-grade astrocytoma. J. Neurooncol..

[6] Gronostajski R.M. (2000). Roles of the NFI/CTF gene family in transcription and development. Gene.

[7] Kruse U., Sippel A.E. (1994). Transcription factor nuclear factor I proteins form stable homo- and heterodimers. FEBS Lett..

[8] Fraser J., Essebier A., Brown A.S., Davila R.A., Harkins D., Zalucki O. (2020). Common regulatory targets of NFIA, NFIX and NFIB during postnatal cerebellar development. Cerebellum.

[9] Adam R.C., Yang H., Ge Y., Infarinato N.R., Gur-Cohen S., Miao Y. (2020). NFI transcription factors provide chromatin access to maintain stem cell identity while preventing unintended lineage fate choices. Nat. Cell Biol..

[10] Wang W., Mullikin-Kilpatrick D., Crandall J.E., Gronostajski R.M., Litwack E.D., Kilpatrick D.L. (2007). Nuclear factor I coordinates multiple phases of cerebellar granule cell development via regulation of cell adhesion molecules. J. Neurosci..

[11] Piper M., Harris L., Barry G., Heng Y.H.E., Plachez C., Gronostajski R.M. (2011). Nuclear factor one X regulates the development of multiple cellular populations in the postnatal cerebellum. J. Comp. Neurol..

[12] Matuzelski E., Bunt J., Harkins D., Lim J.W.C., Gronostajski R.M., Richards L.J. (2017). Transcriptional regulation of *Nfix* by NFIB drives astrocytic maturation within the developing spinal cord. Dev. Biol..

[13] Steele-Perkins G., Plachez C., Butz K.G., Yang G., Bachurski C.J., Kinsman S.L. (2005). The transcription factor gene Nfib is essential for both lung maturation and brain development. Mol. Cell. Biol..

[14] Hsu Y.-C., Osinski J., Campbell C.E., Litwack E.D., Wang D., Liu S. (2011). Mesenchymal nuclear factor I B regulates cell proliferation and epithelial differentiation during lung maturation. Dev. Biol..

[15] Steele-Perkins G., Butz K.G., Lyons G.E., Zeichner-David M., Kim H.-J., Cho M.-I. (2003). Essential role for NFI-C/CTF transcription-replication factor in tooth root development. Mol. Cell. Biol..

[16] Kim T.-H., Bae C.-H., Yang S., Park J.-C., Cho E.-S. (2015). Nfic regulates tooth root patterning and growth. Anat. Cell Biol..

[17] Roh S.Y., Park J.-C. (2017). The role of nuclear factor I-C in tooth and bone development. J. Korean Assoc. Oral Maxillofac. Surg..

[18] Raviram R., Raman A., Preissl S., Ning J., Wu S., Koga T. (2023). Integrated analysis of single-cell chromatin state and transcriptome identified common vulnerability despite glioblastoma heterogeneity. Proc. Natl. Acad. Sci. U. S. A..

[19] Lee J.S., Xiao J., Patel P., Schade J., Wang J., Deneen B. (2014). A novel tumor-promoting role for nuclear factor IA in glioblastomas is mediated through negative regulation of p53, p21, and PAI1. Neuro Oncol..

[20] Stringer B.W., Bunt J., Day B.W., Barry G., Jamieson P.R., Ensbey K.S. (2016). Nuclear factor one B (NFIB) encodes a subtype-specific tumour suppressor in glioblastoma. Oncotarget.

[21] Chen K.-S., Lim J.W.C., Richards L.J., Bunt J. (2017). The convergent roles of the nuclear factor I transcription factors in development and cancer. Cancer Lett..

[22] Osada S., Matsubara T., Daimon S., Terazu Y., Xu M., Nishihara T. (1999). Expression, DNA-binding specificity and transcriptional regulation of nuclear factor 1 family proteins from rat. Biochem. J..

[23] Singh S.K., Wilczynska K.M., Grzybowski A., Yester J., Osrah B., Bryan L. (2011). The unique transcriptional activation domain of nuclear factor-I-X3 is critical to specifically induce marker gene expression in astrocytes. J. Biol. Chem..

[24] Soto L.F., Li Z., Santoso C.S., Berenson A., Ho I., Shen V.X. (2022). Compendium of human transcription factor effector domains. Mol. Cell.

[25] Göös H., Kinnunen M., Salokas K., Tan Z., Liu X., Yadav L. (2022). Human transcription factor protein interaction networks. Nat. Commun..

[26] Deneen B., Ho R., Lukaszewicz A., Hochstim C.J., Gronostajski R.M., Anderson D.J. (2006). The transcription factor NFIA controls the onset of gliogenesis in the developing spinal cord. Neuron.

[27] Kang P., Lee H.K., Glasgow S.M., Finley M., Donti T., Gaber Z.B. (2012). Sox9 and NFIA coordinate a transcriptional regulatory cascade during the initiation of gliogenesis. Neuron.

[28] Glasgow S.M., Zhu W., Stolt C.C., Huang T.-W., Chen F., LoTurco J.J. (2014). Mutual antagonism between Sox10 and NFIA regulates diversification of glial lineages and glioma subtypes. Nat. Neurosci..

[29] Liu X., Salokas K., Weldatsadik R.G., Gawriyski L., Varjosalo M. (2020). Combined proximity labeling and affinity purification−mass spectrometry workflow for mapping and visualizing protein interaction networks. Nat. Protoc..

[30] Campeau E., Ruhl V.E., Rodier F., Smith C.L., Rahmberg B.L., Fuss J.O. (2009). A versatile viral system for expression and depletion of proteins in mammalian cells. PLoS One.

[31] Gawriyski L., Tan Z., Liu X., Chowdhury I., Malaymar Pinar D., Zhang Q. (2024). Interaction network of human early embryonic transcription factors. EMBO Rep..

[32] Langmead B., Salzberg S.L. (2012). Fast gapped-read alignment with Bowtie 2. Nat. Methods.

[33] Danecek P., Bonfield J.K., Liddle J., Marshall J., Ohan V., Pollard M.O. (2021). Twelve years of SAMtools and BCFtools. GigaScience.

[34] Zhang Y., Liu T., Meyer C.A., Eeckhoute J., Johnson D.S., Bernstein B.E. (2008). Model-based analysis of ChIP-seq (MACS). Genome Biol..

[35] Heinz S., Benner C., Spann N., Bertolino E., Lin Y.C., Laslo P. (2010). Simple combinations of lineage-determining transcription factors prime cis-regulatory elements required for macrophage and B cell identities. Mol. Cell.

[36] Ramírez F., Dündar F., Diehl S., Grüning B.A., Manke T. (2014). deepTools: a flexible platform for exploring deep-sequencing data. Nucleic Acids Res..

[37] Yu G., Wang L.-G., Han Y., He Q.-Y. (2012). clusterProfiler: an R package for comparing biological themes among gene clusters. OMICS.

[38] Yu F., Haynes S.E., Teo G.C., Avtonomov D.M., Polasky D.A., Nesvizhskii A.I. (2020). Fast quantitative analysis of timsTOF PASEF data with MSFragger and IonQuant. Mol. Cell. Proteomics.

[39] Teo G., Liu G., Zhang J., Nesvizhskii A.I., Gingras A.-C., Choi H. (2014). SAINTexpress: improvements and additional features in significance analysis of interactome software. J. Proteomics.

[40] Mellacheruvu D., Wright Z., Couzens A.L., Lambert J.-P., St-Denis N.A., Li T. (2013). The CRAPome: a contaminant repository for affinity purification–mass spectrometry data. Nat. Methods.

[41] Huang D.W., Sherman B.T., Lempicki R.A. (2009). Systematic and integrative analysis of large gene lists using DAVID bioinformatics resources. Nat. Protoc..

[42] Mauri M., Elli T., Caviglia G., Uboldi G., Azzi M. (2017). Proceedings of the 12th Biannual Conference on Italian SIGCHI Chapter (ACM, Cagliari Italy).

[43] Tang D., Chen M., Huang X., Zhang G., Zeng L., Zhang G. (2023). SRplot: a free online platform for data visualization and graphing. PLoS One.

[44] Orchard S., Ammari M., Aranda B., Breuza L., Briganti L., Broackes-Carter F. (2014). The MIntAct project—IntAct as a common curation platform for 11 molecular interaction databases. Nucleic Acids Res..

[45] Giurgiu M., Reinhard J., Brauner B., Dunger-Kaltenbach I., Fobo G., Frishman G. (2019). CORUM: the comprehensive resource of mammalian protein complexes—2019. Nucleic Acids Res..

[46] Shannon P., Markiel A., Ozier O., Baliga N.S., Wang J.T., Ramage D. (2003). Cytoscape: a software environment for integrated models of biomolecular interaction networks. Genome Res..

[47] Knight J.D.R., Choi H., Gupta G.D., Pelletier L., Raught B., Nesvizhskii A.I. (2017). ProHits-viz: a suite of web tools for visualizing interaction proteomics data. Nat. Methods.

[48] Abramson J., Adler J., Dunger J., Evans R., Green T., Pritzel A. (2024). Accurate structure prediction of biomolecular interactions with AlphaFold 3. Nature.

[49] Meng E.C., Goddard T.D., Pettersen E.F., Couch G.S., Pearson Z.J., Morris J.H. (2023). UCSF ChimeraX: tools for structure building and analysis. Protein Sci..

[50] Elfmann C., Stülke J. (2023). PAE viewer: a webserver for the interactive visualization of the predicted aligned error for multimer structure predictions and crosslinks. Nucleic Acids Res..

[51] Zhao X., Bitsch S., Kubitz L., Schmitt K., Deweid L., Roehrig A. (2021). ultraID: a compact and efficient enzyme for proximity-dependent biotinylation in living cells. bioRxiv.

[52] Salokas K., Liu X., Öhman T., Chowdhury I., Gawriyski L., Keskitalo S. (2022). Physical and functional interactome atlas of human receptor tyrosine kinases. EMBO Rep..

[53] Liu X., Salokas K., Keskitalo S., Martínez-Botía P., Varjosalo M., Mukhtar S. (2023). Protein-Protein Interactions: Methods and Protocols.

[54] Liu X., Abad L., Chatterjee L., Cristea I.M., Varjosalo M. (2024). Mapping protein-protein interactions by mass spectrometry. Mass Spectrom. Rev..

[55] Lamesch P., Li N., Milstein S., Fan C., Hao T., Szabo G. (2007). hORFeome v3.1: a resource of human open reading frames representing over 10,000 human genes. Genomics.

[56] Ward R.J., Alvarez-Curto E., Milligan G. (2011). Using the Flp-In^TM^ T-Rex^TM^ system to regulate GPCR expression. Methods Mol. Biol..

[57] Liu X., Salokas K., Tamene F., Jiu Y., Weldatsadik R.G., Öhman T. (2018). An AP-MS- and BioID-compatible MAC-tag enables comprehensive mapping of protein interactions and subcellular localizations. Nat. Commun..

[58] Imagawa M., Sakaue R., Tanabe A., Osada S., Nishihara T. (2000). Two nuclear localization signals are required for nuclear translocation of nuclear factor 1-A. FEBS Lett..

[59] Pjanic M., Schmid C.D., Gaussin A., Ambrosini G., Adamcik J., Pjanic P. (2013). Nuclear Factor I genomic binding associates with chromatin boundaries. BMC Genomics.

[60] Strober B.J., Elorbany R., Rhodes K., Krishnan N., Tayeb K., Battle A. (2019). Dynamic genetic regulation of gene expression during cellular differentiation. Science.

[61] Allen B.L., Taatjes D.J. (2015). The mediator complex: a central integrator of transcription. Nat. Rev. Mol. Cell Biol..

[62] Saha D., Animireddy S., Bartholomew B. (2024). The SWI/SNF ATP-dependent chromatin remodeling complex in cell lineage priming and early development. Biochem. Soc. Trans..

[63] Patil A., Strom A.R., Paulo J.A., Collings C.K., Ruff K.M., Shinn M.K. (2023). A disordered region controls cBAF activity via condensation and partner recruitment. Cell.

[64] Quevedo M., Meert L., Dekker M.R., Dekkers D.H.W., Brandsma J.H., van den Berg D.L.C. (2019). Mediator complex interaction partners organize the transcriptional network that defines neural stem cells. Nat. Commun..

[65] Weissmann S., Cloos P.A., Sidoli S., Jensen O.N., Pollard S., Helin K. (2018). The tumor suppressor CIC directly regulates MAPK pathway genes via histone deacetylation. Cancer Res..

[66] Raisch J., Dubois M.-L., Groleau M., Lévesque D., Burger T., Jurkovic C.-M. (2023). Pulse-SILAC and interactomics reveal distinct DDB1-CUL4-associated factors, cellular functions, and protein substrates. Mol. Cell. Proteomics.

[67] Uluca B., Lektemur Esen C., Saritas Erdogan S., Kumbasar A. (2022). NFI transcriptionally represses CDON and is required for SH-SY5Y cell survival. Biochim. Biophys. Acta Gene Regul. Mech..

[68] Piper M., Barry G., Hawkins J., Mason S., Lindwall C., Little E. (2010). NFIA controls telencephalic progenitor cell differentiation through repression of the notch effector Hes1. J. Neurosci..

[69] Piper M., Barry G., Harvey T.J., McLeay R., Smith A.G., Harris L. (2014). NFIB-mediated repression of the epigenetic factor Ezh2 regulates cortical development. J. Neurosci..

[70] Gronostajski R.M. (1987). Site-specific DNA binding of nuclear factor I: effect of the spacer region. Nucleic Acids Res..

[71] Gronostajski R.M. (1986). Analysis of nuclear factor I binding to DNA using degenerate oligonucleotides. Nucleic Acids Res..

[72] Kim B.R., Coyaud E., Laurent E.M.N., St-Germain J., Van de Laar E., Tsao M.-S. (2017). Identification of the SOX2 interactome by BioID reveals EP300 as a mediator of SOX2-dependent squamous differentiation and lung squamous cell carcinoma growth. Mol. Cell. Proteomics.

[73] Denny S.K., Yang D., Chuang C.-H., Brady J.J., Lim J.S., Grüner B.M. (2016). Nfib promotes metastasis through a widespread increase in chromatin accessibility. Cell.

[74] Zhang W., Wang Y., Liu Y., Liu C., Wang Y., He L. (2023). NFIB facilitates replication licensing by acting as a genome organizer. Nat. Commun..

[75] Engelen E., Akinci U., Bryne J.C., Hou J., Gontan C., Moen M. (2011). Sox2 cooperates with Chd7 to regulate genes that are mutated in human syndromes. Nat. Genet..

[76] Moen M.J., Adams H.H.H., Brandsma J.H., Dekkers D.H.W., Akinci U., Karkampouna S. (2017). An interaction network of mental disorder proteins in neural stem cells. Transl. Psychiatry.

[77] Abatti L.E., Lado-Fernández P., Huynh L., Collado M., Hoffman M.M., Mitchell J.A. (2023). Epigenetic reprogramming of a distal developmental enhancer cluster drives SOX2 overexpression in breast and lung adenocarcinoma. Nucleic Acids Res..

[78] Lee D.-S., Song Y.J., Gug H.R., Lee J.-H., Bae H.S., Park J.-C. (2022). Nuclear factor I-C regulates stemness genes and proliferation of stem cells in various mineralized tissue through epithelial-mesenchymal interactions in dental epithelial stem cells. Stem Cell. Int..

[79] Lodato M.A., Ng C.W., Wamstad J.A., Cheng A.W., Thai K.K., Fraenkel E. (2013). SOX2 co-occupies distal enhancer elements with distinct POU factors in ESCs and NPCs to specify cell state. PLoS Genet..

[80] Chen L., Kostadima M., Martens J.H.A., Canu G., Garcia S.P., Turro E. (2014). Transcriptional diversity during lineage commitment of human blood progenitors. Science.

[81] Qiu B., de Vries R.J., Caiazzo M. (2021). Direct cell reprogramming of mouse fibroblasts into functional astrocytes using lentiviral overexpression of the transcription factors NFIA, NFIB, and SOX9. Methods Mol. Biol..

[82] Quist E., Ahlenius H., Canals I. (2021). Transcription factor programming of human pluripotent stem cells to functionally mature astrocytes for monocultures and cocultures with neurons. Methods Mol. Biol..

[83] Shiraishi R., Cancila G., Kumegawa K., Torrejon J., Basili I., Bernardi F. (2024). Cancer-specific epigenome identifies oncogenic hijacking by nuclear factor I family proteins for medulloblastoma progression. Dev. Cell.

[84] Poluben L., Nouri M., Liang J., Varkaris A., Ersoy-Fazlioglu B., Voznesensky O. (2024). Increased chromatin accessibility mediated by nuclear factor I drives transition to androgen receptor splice variant dependence in castration-resistant prostate cancer. bioRxiv.

