# Supplementary material for: Nuclear Factor I Family Members are Key Transcription Factors Regulating Gene Expression
Source: Mol Cell Proteomics. 2024 Nov 29;24(1):100890. doi: 10.1016/j.mcpro.2024.100890 (PMC11775196; doi:10.1016/j.mcpro.2024.100890)
Supplement: Supplemental Data [file mmc12.pdf]

Nuclear Factor I family members are key transcription factors regulating gene expression

Dicle Malaymar Pinar[1,2], Helka Göös[3*], Zenglai Tan[4*], Esa-Pekka Kumpula[1], Iftekhar Chowdhury[1], Zixian Wang[5], Qin Zhang[4], Kari Salokas[1], Salla Keskitalo[1], Gong-Hong Wei[4,5], Asli Kumbasar[2], Markku Varjosalo[1,6§]

[1]Institute of Biotechnology, HiLIFE, University of Helsinki, Helsinki, Finland

[2]Department of Molecular Biology and Genetics, Istanbul Technical University, Istanbul, Turkey

[3]iCell, Research and Development, Finnish Red Cross Blood Service, Helsinki, Finland

[4]Biocenter Oulu and Faculty of Biochemistry and Molecular Medicine, University of Oulu, Oulu, Finland

[5]MOE Key Laboratory of Metabolism and Molecular Medicine & Department of Biochemistry and Molecular Biology of School Basic Medical Sciences, Fudan University Shanghai Cancer Center, Shanghai Medical College of Fudan University, Shanghai, China

[6]iCAN Digital Precision Cancer Medicine Flagship, University of Helsinki, Helsinki, Finland

*Equal contribution

§ Correspondence: Markku Varjosalo, markku.varjosalo@helsinki.fi

Running title: A Multiomics Study of NFI Transcription Factor Networks

Table S1. NFIs DBD regions and target sequences from ChIP-Seq hits used for AlphaFold3 predictions.

**POI**	**Predicted DNA Binding and dimerization regions**	**Target Sequences**
**NFIA**	1-245 aa	5’GCCAGCCTGGCCCAGAAGCCTCTGCCTG-3’<br>3’CAGGCAGAGGCTTCTGGGCCAGGCTGGC-5’
**NFIB**	15-235 aa	5’TGTAACTTGGCTGCCCGCCAGAGGCCT-3’<br>3’AGGCCTCTGGCGGGCAGCCAAGTTACA-5’
**NFIC**	14-261 aa	5’CTGGCGGCCAGCTCAGGATGCCAGGCGGC-3’<br>3’GCCGCCTGGCATCCTGAGCTGGCCGCCAG-5’
**NFIX**	1-249 aa	5’CATGGTTGGCACCCTGCCAGCTGG-3’<br>3’CCAGCTGGCAGGGTGCCAACCATG-5’

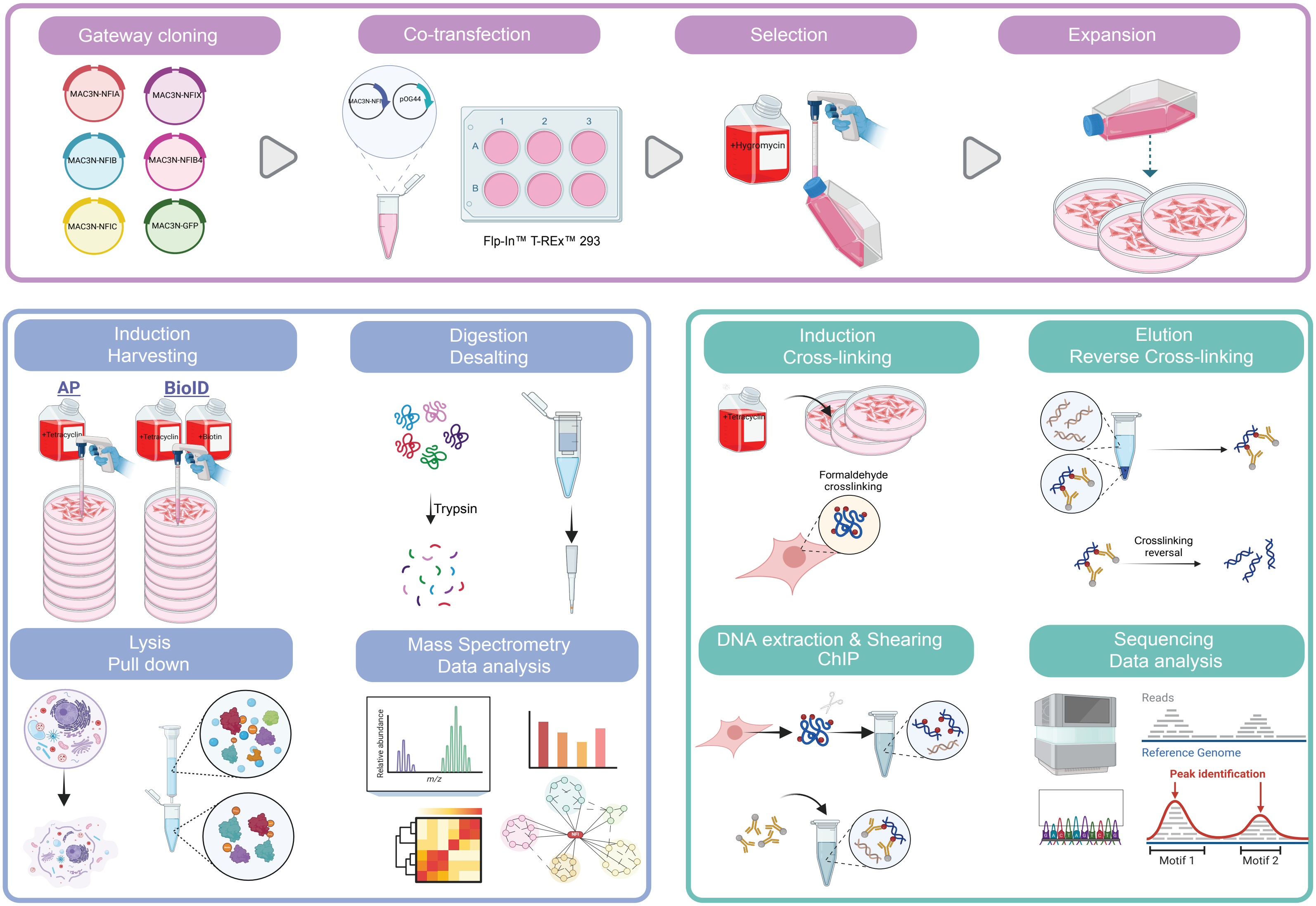
Gateway cloning
MAC3N-NFIA
MAC3N-NFIX
MAC3N-NFIB
MAC3N-NFIB4
MAC3N-NFIC
MAC3N-GFP
Co-transfection
pOG44
Flp-In™ T-REx™ 293
Selection
+Hygromycin
Expansion
Induction
Harvesting
AP
BioID
+Tetracyclin
+Biotin
Digestion
Desalting
Trypsin
Lysis
Pull down
Mass Spectrometry
Data analysis
Relative abundance
m/z
Induction
Cross-linking
Formaldehyde crosslinking
Elution
Reverse Cross-linking
Crosslinking reversal
DNA extraction & Shearing
ChIP
Sequencing
Data analysis
Reads
Reference Genome
Peak identification
Motif 1
Motif 2

Figure S1. Experimental workflow for analyzing interactome and targetome of NFI TFs. Schematic illustration of experimental workflow employed this study. NFIs were tagged N-terminally with a MAC3-tag and co-transfected with pOG44 to generate inducible and isogenic Flp-in TRex 293 cell lines. After hygromycin selection and expansion cells were induced by tetracycline containing media 24 hours, for the BioID experiment, cells supplemented with biotin containing media for 16 hours before harvesting. Induced cells were used for AP-MS, BioID and ChIP sequencing experiments. AP-MS and BioID samples purified, digested into peptides analysed by LC-MS/MS. ChIP-seq samples precipitated with HA antibody prior to sequencing. Raw files were later analysed with several bioinformatic methods.

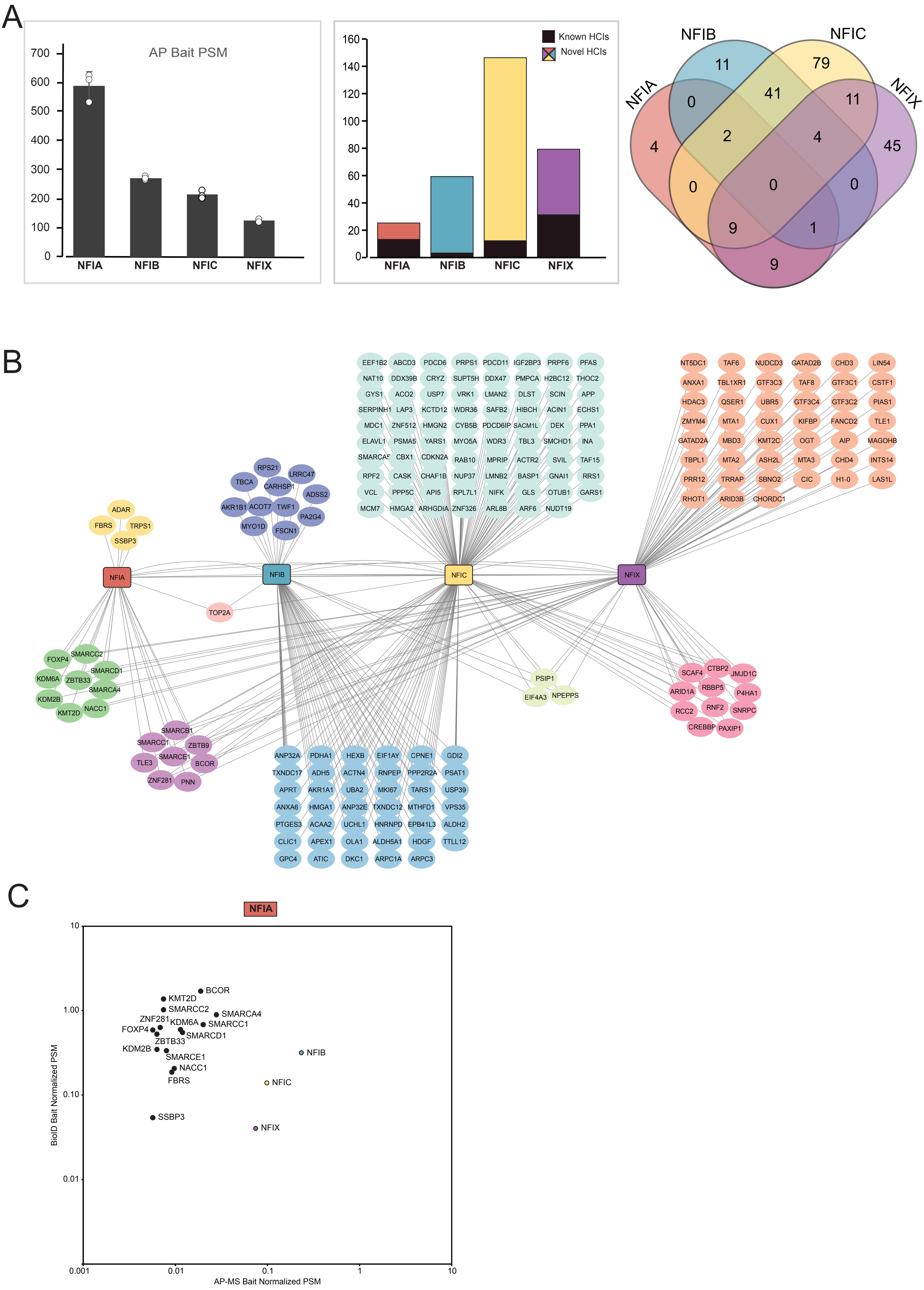
A
AP Bait PSM
700
600
500
400
300
200
100
0
NFIA
NFIB
NFIC
NFIX
160
140
120
100
80
60
40
20
0
Known HCIs
Novel HCIs
NFIA
NFIB
NFIC
NFIX
NFIA
NFIB
NFIC
NFIX
11
79
0
41
11
4
2
4
45
0
0
0
9
1
9
B
EEF1B2 ABCD3 PDCD6 PRPS1 PDCD11 IGF2BP3 PRPF6 PFAS
NAT10 DDX39B CRYZ SUPT5H DDX47 PMPCA H2BC12 THOC2
GYS1 ACO2 USP7 VRK1 LMAN2 DLST SCIN APP
SERPINH1 LAP3 KCTD12 WDR36 SAFB2 HIBCH ACIN1 ECHS1
MDC1 ZNF512 HMGN2 CYB5B PDCD6IP SACM1L DEK PPA1
ELAVL1 PSMA5 YARS1 MYO5A WDR3 TBL3 SMCHD1 INA
CBX1 CDKN2A RAB10 MPRIP ACTR2 SVIL TAF15
RPF2 CASK CHAF1B NUP37 LMNB2 BASP1 GNAI1 RRS1
VCL PPP5C API5 RPL7L1 NIFK GLS OTUB1 GARS1
MCM7 HMGA2 ARHGDIA ZNF326 ARL8B ARF6 NUDT19
NT5DC1 TAF6 NUDCD3 GATAD2B CHD3 LIN54
ANXA1 TBL1XR1 GTF3C3 TAF8 GTF3C1 CSTF1
HDAC3 QSER1 UBR5 GTF3C4 GTF3C2 PIAS1
ZMYM4 MTA1 CUX1 KIFBP FANCD2 TLE1
GATAD2A MBD3 KMT2C OGT AIP MAGOHB
TBPL1 MTA2 ASH2L MTA3 CHD4 INTS14
PRR12 TRRAP SBNO2 CIC H1-0 LAS1L
RHOT1 ARID3B CHORDC1
RPS21 LRRC47 TBCA CARHSP1 ADSS2 AKR1B1 ACOT7 TWF1 PA2G4 MYO1D FSCN1
ADAR FBRS TRPS1 SSBP3
NFIA
NFIB
NFIC
NFIX
TOP2A
FOXP4 SMARCC2 SMARCD1 KDM6A ZBTB33 SMARCA4 KDM2B NACC1 KMT2D
SMARCB1 SMARCC1 ZBTB9 SMARCE1 TLE3 BCOR ZNF281 PNN
PSIP1 EIF4A3 NPEPPS
SCAF4 CTBP2 JMJD1C ARID1A RBBP5 P4HA1 RCC2 RNF2 SNRPC CREBBP PAXIP1
ANP32A PDHA1 HEXB EIF1AY CPNE1 GDI2
TXNDC17 ADH5 ACTN4 RNPEP PPP2R2A PSAT1
APRT AKR1A1 UBA2 MKI67 TARS1 USP39
ANXA6 HMGA1 ANP32E TXNDC12 MTHFD1 VPS35
PTGES3 ACAA2 UCHL1 HNRNPD EPB41L3 ALDH2
CLIC1 APEX1 OLA1 ALDH5A1 HDGF TTLL12
GPC4 ATIC DKC1 ARPC1A ARPC3
C
NFIA
10
1.00
0.10
0.01
BioID Bait Normalized PSM
BCOR
KMT2D
SMARCC2
SMARCA4
ZNF281
KDM6A
SMARCC1
FOXP4
SMARCD1
ZBTB33
KDM2B
SMARCE1
NFIB
NACC1
FBRS
NFIC
SSBP3
NFIX
0.001
0.01
0.1
1
10
AP-MS Bait Normalized PSM

Figure S2. Affinity purification data analysis. A) Average number of NFI Bait PSM of the AP experiment replicates. Number of known and novel high-confidence protein interactors of NFI proteins and comparison of the HCI lists by Venn diagram. **B)** Visualization of the stable interactions network of NFI members revealed by affinity purification experiment. **C)** Characterization of interaction distances based on Bait Normalized PSM values of common HCIs in AP-MS and BioID data.

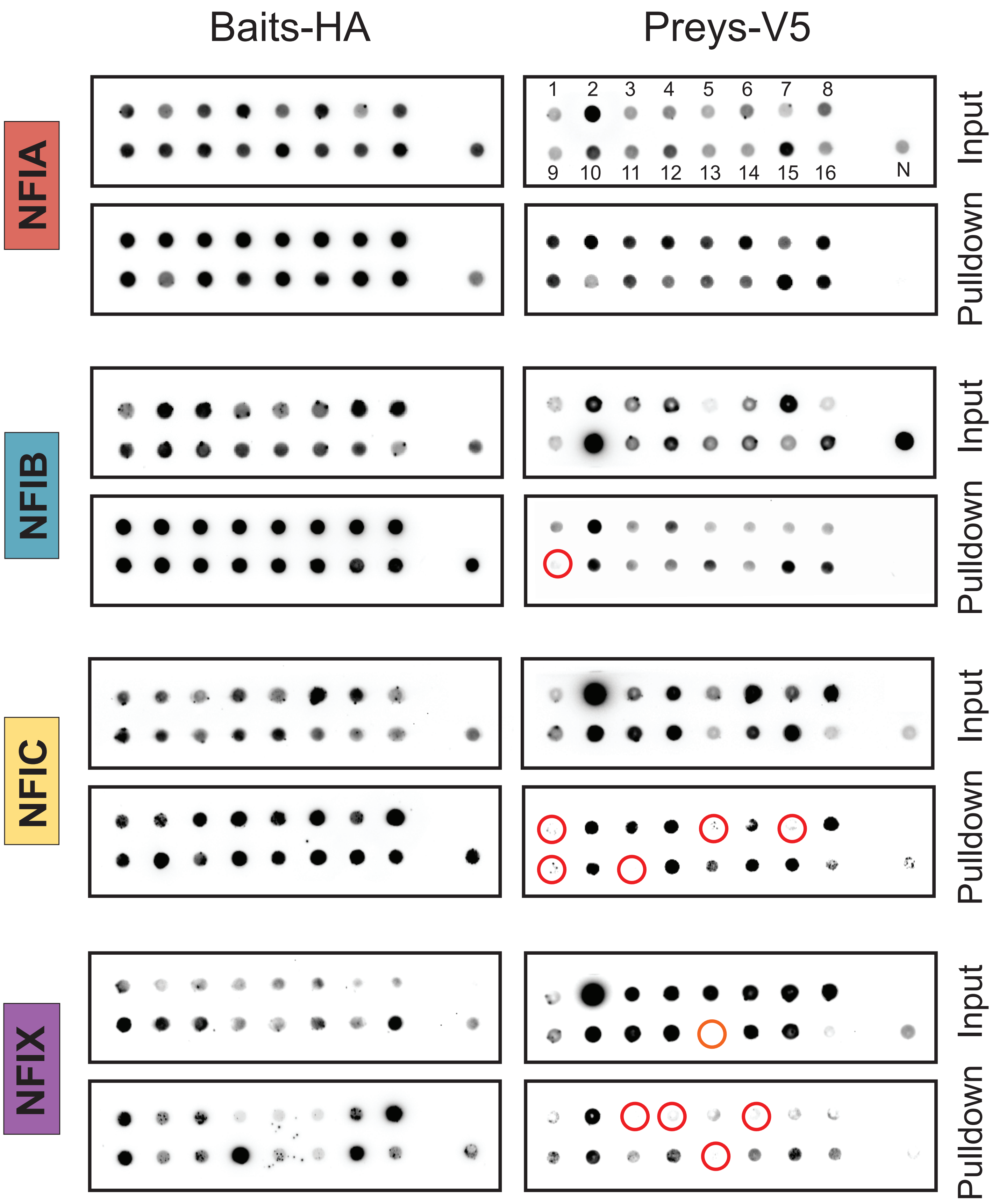

Baits-HA
Preys-V5
NFIA
NFIB
NFIC
NFIX
Input
Pulldown
1 2 3 4 5 6 7 8
9 10 11 12 13 14 15 16
N

Figure S3. Validation of NFI-SWI/SNF protein interaction data by co-expression, pull down and dot blotting. NFI-SWI/SNF protein interaction data were evaluated by affinity pull down and dot blotting, related to Dataset 11. In total, 64 selected interaction pairs from analyzed using co-expression, of which 54 pairs (84%) showed positive signal. Negative signals are shown in red circles. A prey protein with no visible expression is shown in orange circle on the input blot. The NFI bait proteins tagged with a Strep-HA and SWI/SNF prey proteins tagged with V5 were coexpressed in HEK293 cells. Strep-HA-tagged proteins were immunoprecipitated with the Strep-Tactin Sepharose resin, then the immunoprecipitated complexes were analyzed by dot blotting with anti-HA (rabbit) antibody and anti-V5 (mouse) antibody, respectively. Five percent of the total cell lysate was used as the input loading control. The prey proteins are labeled numerically in the figure: 1-BRD9, 2-SMARCC2, 3-BICRA, 4-SMARCA2, 5-BCL7C, 6-DPF3, 7-BCL7A, 8-SMARCD1, 9-ARID2, 10-SMARCB1, 11-DPF2, 12-SMARCD3, 13-BICRAL, 14-SS18L1, 15-DPF1, 16-SMARCE1, N-LacZ.

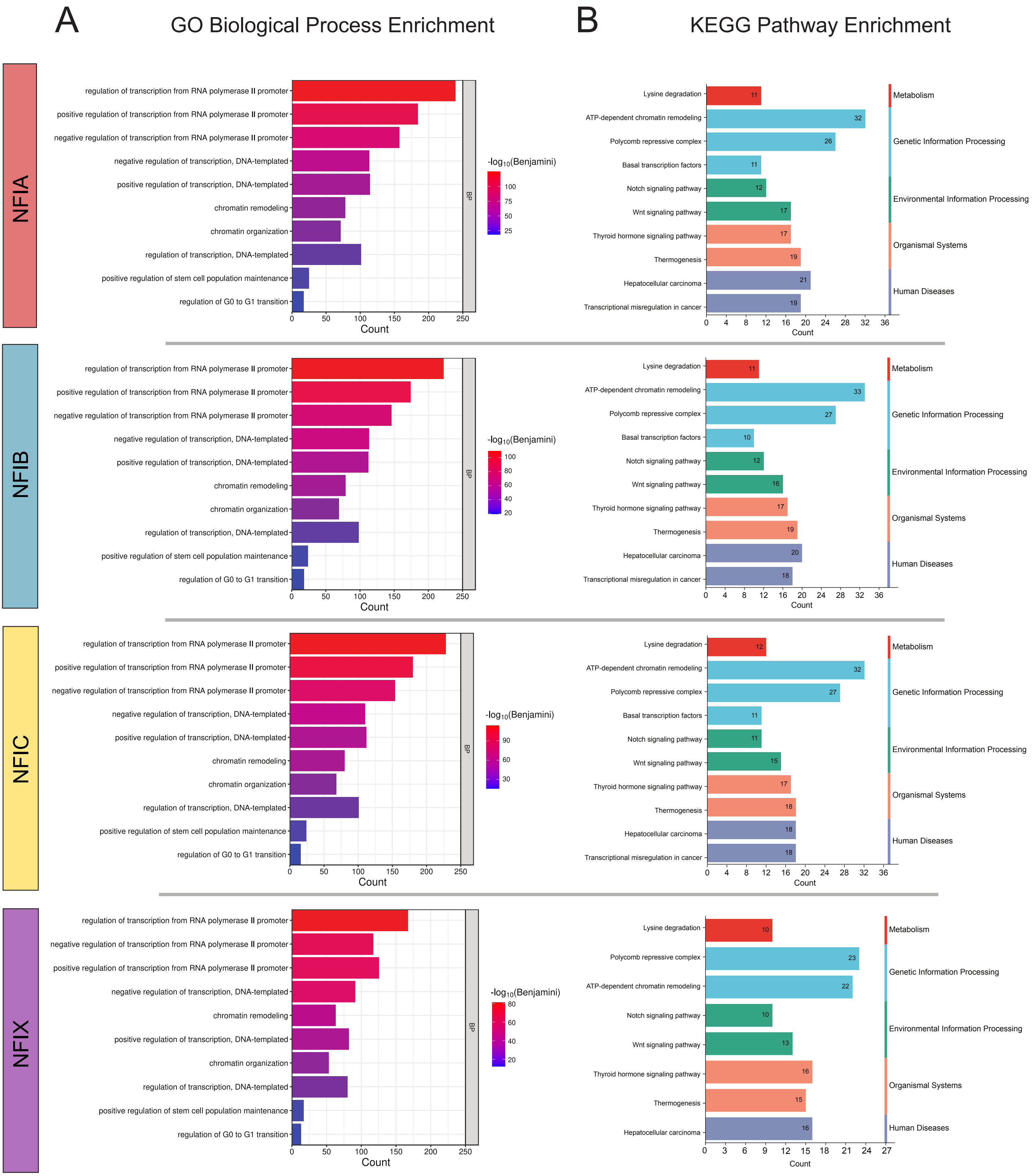

A
GO Biological Process Enrichment
B
KEGG Pathway Enrichment
NFIA
NFIB
NFIC
NFIX
regulation of transcription from RNA polymerase II promoter
positive regulation of transcription from RNA polymerase II promoter
negative regulation of transcription from RNA polymerase II promoter
negative regulation of transcription, DNA-templated
positive regulation of transcription, DNA-templated
chromatin remodeling
chromatin organization
regulation of transcription, DNA-templated
positive regulation of stem cell population maintenance
regulation of G0 to G1 transition
BP
Count
-log10(Benjamini)
Lysine degradation
ATP-dependent chromatin remodeling
Polycomb repressive complex
Basal transcription factors
Notch signaling pathway
Wnt signaling pathway
Thyroid hormone signaling pathway
Thermogenesis
Hepatocellular carcinoma
Transcriptional misregulation in cancer
Metabolism
Genetic Information Processing
Environmental Information Processing
Organismal Systems
Human Diseases

Figure S4. Gene Ontology Biological Process (GO-BP) and KEGG Pathway enrichment analysis of HCIs of NFIs. A) GO Biological process (GO-BP) enrichment analysis of NFI HCI proteins. Most significant first 10 biological process shown in the graphs. Size of bar graphs shows the count of interactors related with the processes. Color of bars the represent the adjusted-p values (Benjamini). **B)** KEGG pathway enrichment analysis of NFI HCI proteins. Pathways with Benjamini<0.001 shown in the figure. Size of bar graphs shows the count of interactors related with the pathways.

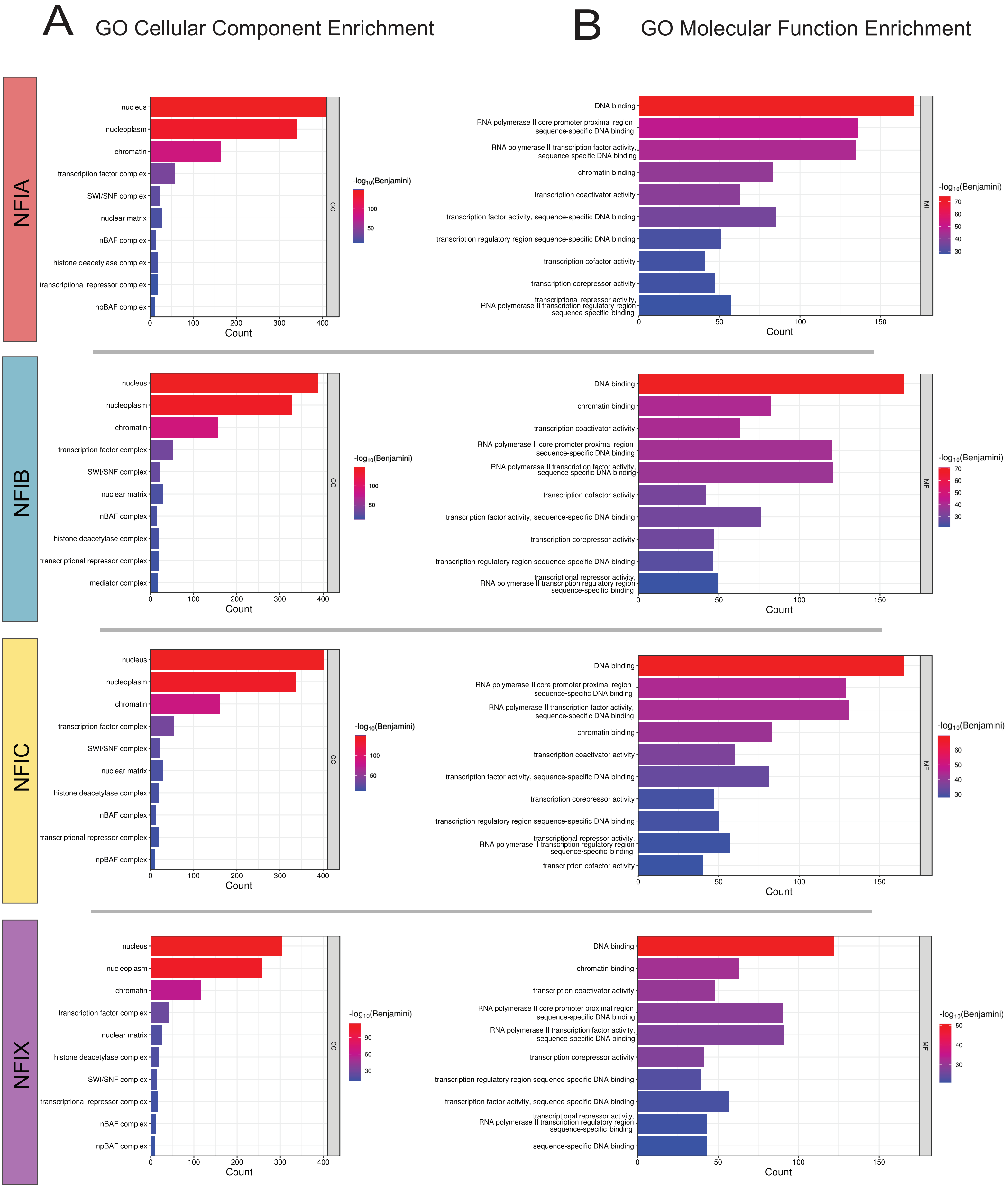

A
GO Cellular Component Enrichment
B
GO Molecular Function Enrichment
NFIA
nucleus
nucleoplasm
chromatin
transcription factor complex
SWI/SNF complex
nuclear matrix
nBAF complex
histone deacetylase complex
transcriptional repressor complex
npBAF complex
Count
CC
$-\log_{10}$(Benjamini)
DNA binding
RNA polymerase II core promoter proximal region sequence-specific DNA binding
RNA polymerase II transcription factor activity, sequence-specific DNA binding
chromatin binding
transcription coactivator activity
transcription factor activity, sequence-specific DNA binding
transcription regulatory region sequence-specific DNA binding
transcription cofactor activity
transcription corepressor activity
transcriptional repressor activity, RNA polymerase II transcription regulatory region sequence-specific binding
MF
NFIB
mediator complex
NFIC
NFIX
sequence-specific DNA binding

Figure S5. Gene Ontology Cellular Component (GO-CC) and Molecular Function (GO-MF) enrichment analysis of HCIs of NFIs. A) GO Cellular Component (GO-CC) enrichment analysis of NFI HCI proteins identified 16h BioID experiment. Most significant first 10 terms shown in the graphs. Size of bar graphs shows the count of interactors related with that term. Color of bars the represent the adjusted-p values (Benjamini). **B)** GO Molecular Function (GO-MF) enrichment analysis of NFI HCI proteins identified 16h BioID experiment. Most significant first 10 terms shown in the graphs. Size of bar graphs shows the count of interactors related with that term. Color of bars the represent the adjusted-p values (Benjamini).

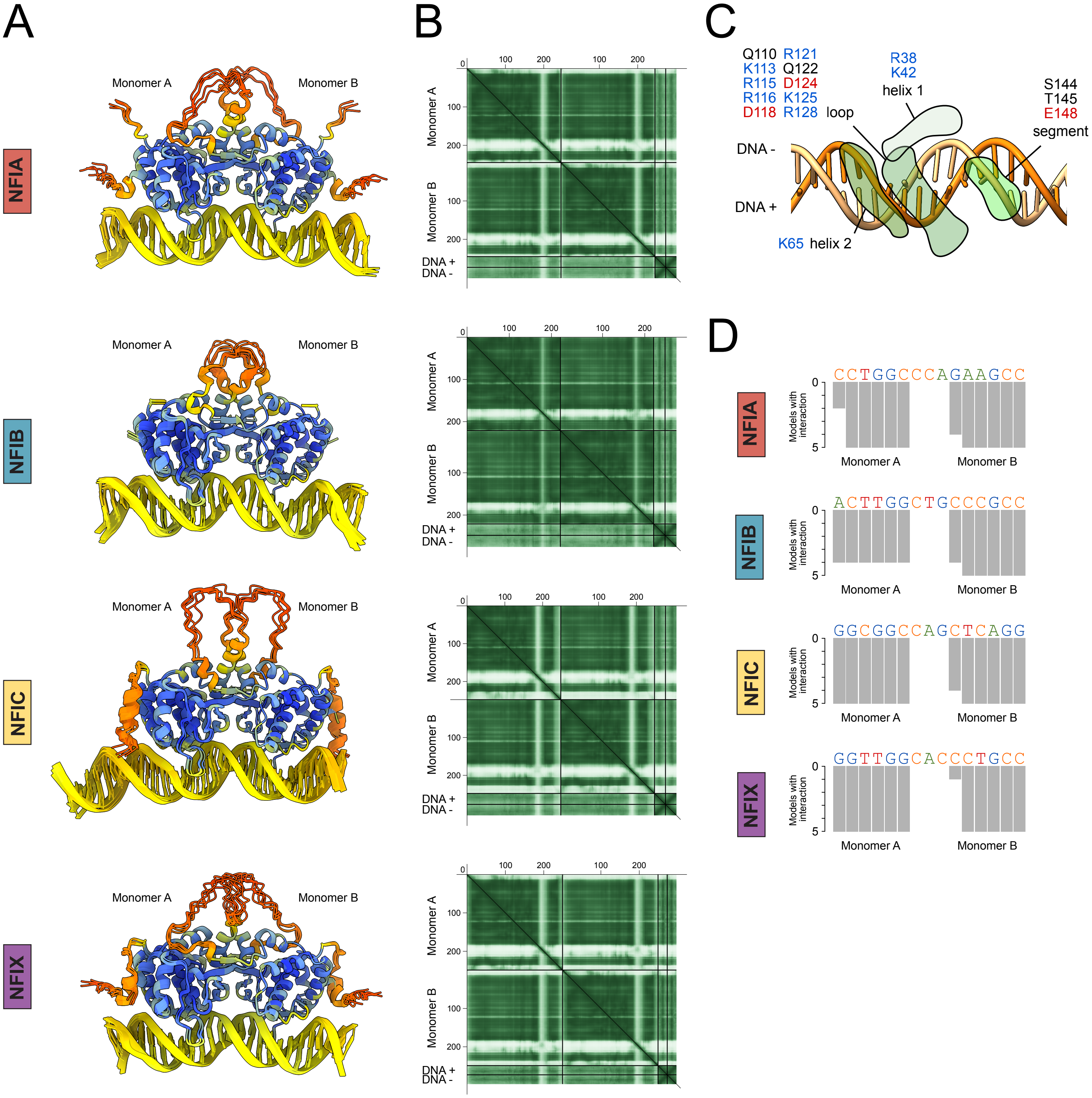

A
B
C
D
NFIA
NFIB
NFIC
NFIX
Monomer A
Monomer B
DNA +
DNA -
Q110 R121
K113 Q122
R115 D124
R116 K125
D118 R128 loop
R38
K42
helix 1
S144
T145
E148
segment
K65 helix 2
CCTGGCCCAGAAGCC
ACTTGGCTGCCCGCC
GGCGGCCAGCTCAGG
GGTTGGCACCCTGCC
Models with interaction

Figure S6. Structural predictions and binding residue analysis of NFIs. A) AlphaFold3 predictions of the NFIs in complex with DNA duplex identified by ChIP-Seq. All 5 models from a prediction run are shown superposed, colored by the per-residue pLDDT score. **B)** Predicted alignment error plots of the highest confidence model for each NFI. **C)** Schematic of binding residues identified in NFIs based on the models. The interacting residues of the NFIs are identical to those of NFIA shown in the schematic. Positively charged amino acids are shown in blue, negatively charged amino acids in red. **D)** Binding DNA sequences identified from the predictions and the binding residues of NFIA shown as a schematic. The DNA-protein interactions identified by the "interfaces" routine in ChimeraX were counted from each prediction, shown as bar plots.

A

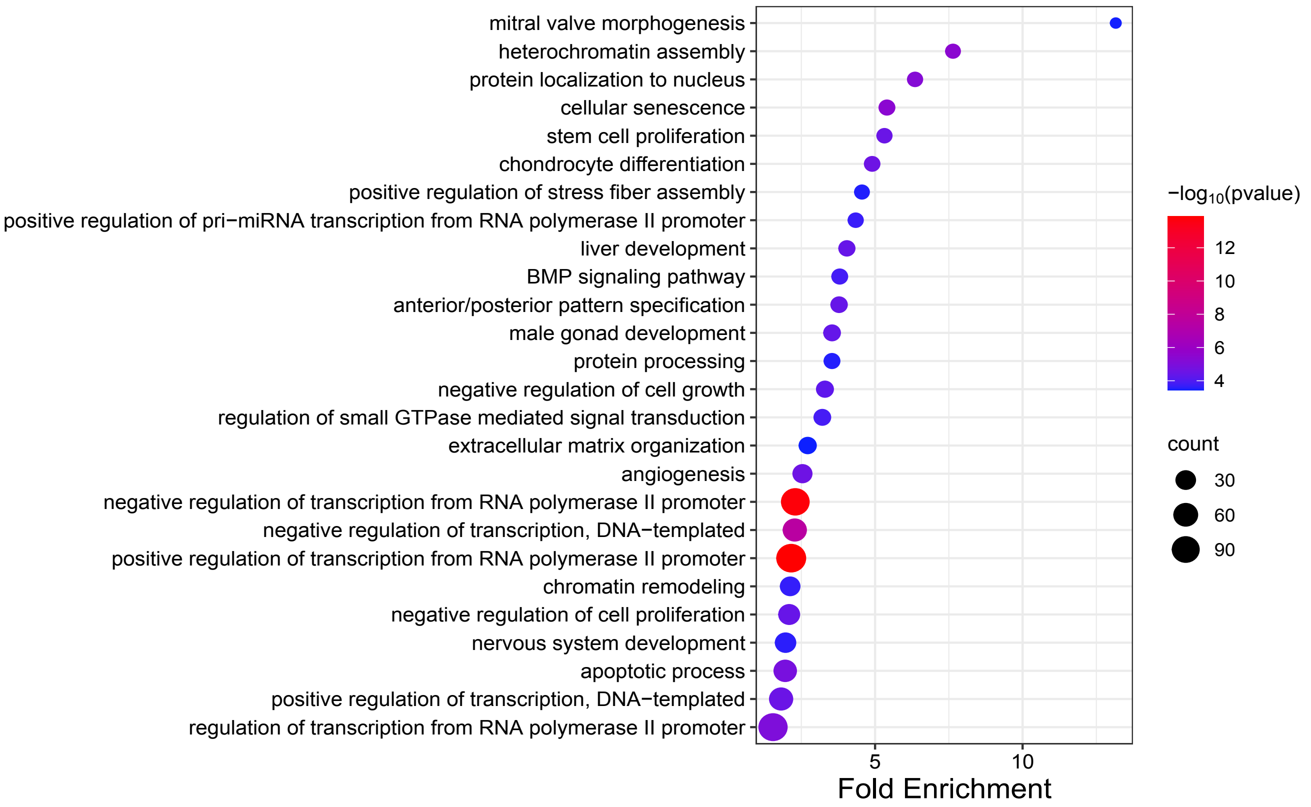

mitral valve morphogenesis
heterochromatin assembly
protein localization to nucleus
cellular senescence
stem cell proliferation
chondrocyte differentiation
positive regulation of stress fiber assembly
positive regulation of pri−miRNA transcription from RNA polymerase II promoter
liver development
BMP signaling pathway
anterior/posterior pattern specification
male gonad development
protein processing
negative regulation of cell growth
regulation of small GTPase mediated signal transduction
extracellular matrix organization
angiogenesis
negative regulation of transcription from RNA polymerase II promoter
negative regulation of transcription, DNA−templated
positive regulation of transcription from RNA polymerase II promoter
chromatin remodeling
negative regulation of cell proliferation
nervous system development
apoptotic process
positive regulation of transcription, DNA−templated
regulation of transcription from RNA polymerase II promoter
5
10
Fold Enrichment
−log10(pvalue)
12
10
8
6
4
count
30
60
90

B

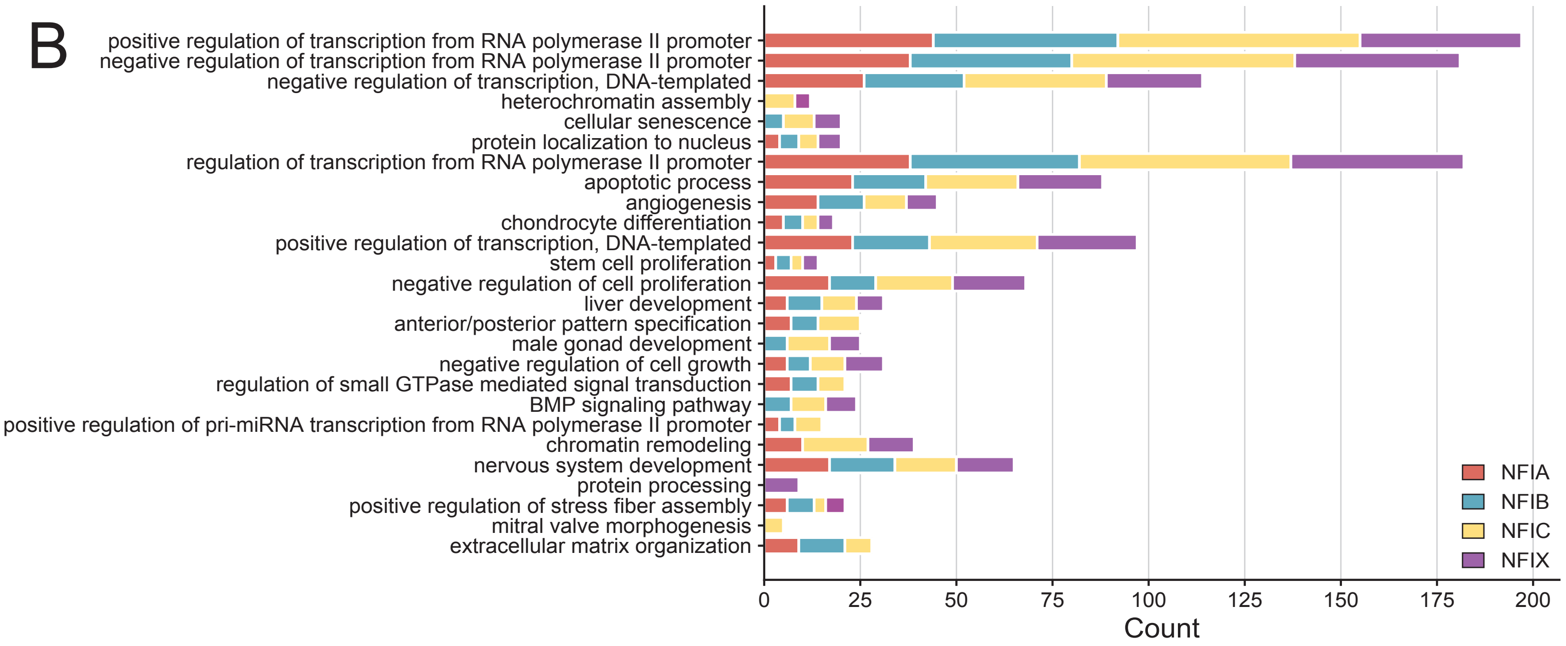

positive regulation of transcription from RNA polymerase II promoter
negative regulation of transcription from RNA polymerase II promoter
negative regulation of transcription, DNA-templated
heterochromatin assembly
cellular senescence
protein localization to nucleus
regulation of transcription from RNA polymerase II promoter
apoptotic process
angiogenesis
chondrocyte differentiation
positive regulation of transcription, DNA-templated
stem cell proliferation
negative regulation of cell proliferation
liver development
anterior/posterior pattern specification
male gonad development
negative regulation of cell growth
regulation of small GTPase mediated signal transduction
BMP signaling pathway
positive regulation of pri-miRNA transcription from RNA polymerase II promoter
chromatin remodeling
nervous system development
protein processing
positive regulation of stress fiber assembly
mitral valve morphogenesis
extracellular matrix organization
0
25
50
75
100
125
150
175
200
Count
NFIA
NFIB
NFIC
NFIX

Figure S7. Gene Ontology Biological Process (GO-BP) enrichment analysis of NFI target genes. A) The GO-BP enrichment analysis of all NFIs target genes detected by ChIP-Seq. GO-BP terms are considered as enriched according to Benjamini values (adjusted p value) less than 0.05. Dot size represents the number of target genes involved in each biological process and shown in left panel together with color scale bar of -log10(p-adjusted). **B)** Stacked bar graph showing gene counts of enriched biological process terms for each NFI members.
